# Flexural Strengthening of Reinforced Concrete Beams Using Textile-Reinforced Mortar, Carbon Fiber-Reinforced Polymer Systems and Near-Surface-Mounted Composites: An Experimental Comparison

**DOI:** 10.3390/ma19173660

**Published:** 2026-08-28

**Authors:** Ali Hussein Hadi Hadi, Mehmet Baran, Sercan Tuna Akkaya, Nooruldeen Tareq Khalid Khalid, Mustafa Khalid Abdullah Alageedi, Faruk Eren Erdoğdu, Özgür Anıl, İrfan Kocaman, Ömer Mercimek

**Affiliations:** 1 Department of Civil Engineering, Ankara Yıldırım Beyazıt University, 06010 Ankara, Türkiye; alihussein.dulaimi@gmail.com (A.H.H.H.); mehmet.baran@aybu.edu.tr (M.B.); sercantuna.akkaya@aybu.edu.tr (S.T.A.); nooruldeentareq@gmail.com (N.T.K.K.); mustafa.alageedi@gmail.com (M.K.A.A.); 2 Department of Civil Engineering, Ankara University, 06830 Ankara, Türkiye; farukerenerd@hotmail.com; 3 Department of Civil Engineering, Gazi University, 06570 Ankara, Türkiye; oanil@gazi.edu.tr; 4 Department of Civil Engineering, Erzurum Technical University, 25050 Erzurum, Türkiye; irfan.kocaman@erzurum.edu.tr

**Keywords:** reinforced concrete beams, flexural strengthening, CFRP, TRM, NSM

## Abstract

This study conducted an experimental investigation of the flexural behavior of reinforced concrete beams strengthened using externally bonded and near-surface-mounted techniques. The strengthening materials consisted of carbon fiber-reinforced polymer (CFRP) sheets, carbon fiber plates, and textile-reinforced mortar (TRM) with carbon, basalt, and glass textiles, as well as near-surface-mounted (NSM) glass and carbon bars. Twenty-two reinforced concrete beams were tested, comprising one reference specimen and 21 strengthened specimens with different strengthening techniques, numbers of layers, and anchorage conditions. The results showed a marked improvement in the flexural performance of the strengthened specimens compared with the reference specimen, which had an ultimate load capacity of 60.01 kN. CFRP strengthening achieved the highest increase in ultimate load capacity and stiffness, with the best specimen recording 110.44 kN and a stiffness value of 18.83 kN/mm. Carbon fiber plates also exhibited a significant improvement in strength. In contrast, TRM and NSM systems produced comparatively lower strength gains but offered superior ductility and more gradual failure behavior. In terms of TRM strengthening, carbon textiles demonstrated the highest ultimate load capacity, whereas basalt and glass textiles enhanced deformation capacity. The use of fan-type anchors further enhanced the efficiency of externally bonded strengthening systems by delaying premature debonding. Within the scope of the present experimental program, the findings provide a direct comparison of the investigated carbon fiber-reinforced polymer (CFRP), carbon-plate (CP), textile-reinforced mortar (TRM), and near-surface-mounted (NSM) strengthening configurations under identical testing conditions and demonstrate their relative effects on strength, stiffness, ductility, energy dissipation, and failure behavior. These results provide comparative experimental evidence that may assist in selecting strengthening strategies for RC beams with comparable characteristics; however, broader generalization requires consideration of additional member geometries, material properties, reinforcement configurations, and loading conditions.

## 1. Introduction

The need to strengthen existing concrete elements is constantly increasing due to progressive deterioration during service life, inadequate maintenance, and the necessity to comply with modern design requirements. Due to the high costs, replacing worn-out concrete elements with new ones is not a viable option and is generally not considered economical. Consequently, the focus in the construction sector has shifted from new construction to the renovation and strengthening of existing structural elements. The restoration of existing structures now accounts for approximately 50% of the total construction sector [1].

Over the past three decades, fiber-reinforced polymer (FRP) has progressively become the favored option for enhancing concrete and masonry structures, outpacing traditional reinforcing techniques such as steel or reinforced concrete jacketing. This preference largely comes from the advantageous properties of FRP, particularly its corrosion resistance, high strength-to-weight ratio, ease of application, and minimal effect on structural geometry. National and international regulations for the design of elements reinforced with Externally Bonded Reinforcements (EBRs) using FRP are being issued globally (ACI Committee 440.2R [2]; Fib Bulletin 14 [3]; ISIS Canada [4]; Japan Society of Civil Engineers [5]; CNR DT200 [6]).

About a decade ago, a new cement-based composite material known as textile-reinforced mortar (TRM) was invented to strengthen and repair concrete structures [7] as an alternative to FRP, aiming to overcome some of its limitations. Since its first appearance, this material has garnered significant attention in the engineering community. Textile-reinforced mortar (TRM) is a composite material composed of fiber rovings—usually fabricated from carbon, basalt, or glass—configured as textiles and embedded into inorganic matrices like cement-based mortars [8]. The terminology “textile concrete” is most commonly used in situations where this technique is an alternative to reinforced concrete, with traditional steel strengthening being replaced by textile. This replacement considerably reduces the size of the cover and the thickness of the structural elements. Research assessing the application of textile concrete in strengthening and repair [9] frequently employs varied terminology for the approach, such as fabric-reinforced cementitious matrix (FRCM) [10], textile-reinforced mortar (TRM) [11], or fabric-reinforced mortar (FRM) [12]. TRM is regarded as a cost-effective solution, as mortars are typically less expensive than epoxy resins. It provides numerous practical benefits: compatibility with concrete and masonry substrates, safety and ease of handling for workers, applicability in low temperatures or on damp surfaces, and resistance to elevated temperatures [13].

The TRM offers several advantages among alternative strengthening methods: it is applied in thin layers, which minimally alters the element’s cross-section; the cementitious matrix enhances adhesion, is compatible with concrete substrates and wet surfaces, and contributes to a more uniform repaired structure. Additionally, the textile strengthening embedded in a cementitious matrix exhibits superior performance at elevated temperatures compared to resin-impregnated meshes [14]. Furthermore, the method avoids metallic strengthening, rendering it an extremely advantageous option, particularly in harsh environments in which corrosion is prevalent.

The near surface mounted (NSM) technique employing fiber-reinforced polymer (FRP) strengthening has emerged as a promising approach for enhancing reinforced concrete (RC) elements and masonry. This process involves the installation of FRP strengthening into slits cut into the concrete body, utilizing cement mortar or epoxy as bonding agents. Its application offers numerous benefits compared to the externally bonded (EB) FRP method: enhanced protection, superior bonding, improved aesthetics, and surface preparation [15]. Despite the enhanced performance of bonds relative to the EB system, they remain a critical consideration in the design of NSM FRP-strengthened elements. The bond at the two NSM interfaces (FRP–adhesive, adhesive–concrete) is influenced by FRP characteristics, FRP surface treatment, bar dimensions, groove surface, groove geometry, adhesive, test configuration, and concrete parameters [16].

Most often, concrete elements fail because of the compaction of the concrete or the yielding of the internal reinforcing steel. FRP materials exhibit linear elastic behavior until failure; however, their great strength is typically not realized when employed as external strengthening, as the reinforced elements frequently break due to a mechanism termed debonding. This failure pattern is dependent upon the bond characteristics at the concrete–FRP strengthening interface and often occurs with the separation of a concrete cover of variable thickness. The failure location along the beam and the thickness of the detached concrete primarily depend on the cracking pattern, the amount of internal steel strengthening, the number of steel stirrups, the loading system, and the relationship between shear and normal bond stresses at the interfaces [17,18]. The ductility of EBR-reinforced concrete members mostly depends on the failure modes that may arise. End anchorage systems may be highly beneficial in preventing or postponing debonding failures, hence significantly enhancing strength and ductility.

Many studies have provided further insights into the behavior of FRP-strengthened RC beams. Esfahani et al. [19] examined the flexural performance of reinforced concrete beams enhanced with externally bonded carbon fiber-reinforced polymer (CFRP) sheets, specifically analyzing the impact of the tensile reinforcing bar ratio on strengthening efficacy, ductility, and failure mode. CFRP was implemented on the soffit following surface preparation, with certain sheets extending beyond the supports to avert premature end debonding. The strengthened beams exhibited significant enhancements in stiffness and ultimate load-bearing capacity relative to the control specimens. The load–deflection curves indicated that CFRP strengthening improved post-cracking stiffness and delayed the yielding of tensile steel. In another study, Mohammed et al. [20] offered an extensive experimental and analytical examination of the flexural performance of reinforced concrete T-beams enhanced with hybrid Aluminum Alloy (AA) and Carbon Fiber-Reinforced Polymer (CFRP) systems. All specimens were subjected to four-point bending tests to determine their structural performance. The experimental results indicated that all specimens showed flexural failure modes, with laminate debonding being determined to be the primary failure mechanism. The strengthened beams exhibited significant enhancements in load-carrying capacity, with improvements between 18% and 77% for specimens with low reinforcement ratios and 10% to 44% for those with high reinforcement ratios. Mercimek et al. [21] performed a comprehensive experimental and numerical analysis of the seismic performance of shear-deficient reinforced concrete (RC) beams strengthened with externally bonded CFRP strips utilizing fan-type anchors. For beams consisting of 25 MPa concrete, strengthening enhanced the maximum load by approximately 20%, initial stiffness by 12%, displacement ductility by 304%, and cumulative energy dissipation by 453% in comparison to the shear-deficient reference beam. Correspondingly, for beams with 10 MPa concrete, the strengthened specimens exhibited average enhancements of 12%, 22%, 237%, and 381%, respectively. The width of the strip had a minimal effect on strength but significantly increased ductility and energy dissipation, while reducing the strip spacing (150 mm compared to 250 mm) substantially improved stiffness, ductility, and cumulative energy dissipation. Assad et al. [22] conducted a comprehensive study on the bending behavior of reinforced concrete beams strengthened with CFRP sheets and reinforced using CFRP spike anchors to reduce some common problems, such as premature debonding of the FRP sheets. The load deflection data indicate significant enhancements in stiffness and strength for all reinforced beams, with anchored specimens exhibiting 60–104% increases in ultimate load capacity compared to the control beam and 14–28% increases compared to the unanchored strengthened beam. Their study indicates that CFRP anchors significantly improve the flexural performance of strengthened RC beams by postponing debonding, increasing CFRP strain utilization, and enhancing load capacity. Ceroni [23] performed a comprehensive study of the structural performance of reinforced concrete (RC) beams enhanced with fiber-reinforced polymer (FRP) materials, contrasting externally bonded reinforcement (EBR) utilizing carbon FRP sheets with near-surface-mounted (NSM) carbon bars. The results indicated that EBR strengthening greatly enhanced load-carrying capacity, with increases of roughly 26–50% in beams with lower internal steel ratios and 17–33% in those that had higher steel content.

Several studies have also presented the behavior of reinforced concrete beams using TRM technology. Raoof et al. [24] performed a comprehensive laboratory comparison between textile-reinforced mortar (TRM) and fiber-reinforced polymer (FRP) systems for the flexural strengthening of reinforced concrete (RC) beams. Their results indicated that both methods markedly improved flexural capacity, stiffness, and post-yielding behavior compared to the control beam, with flexural strength enhancements ranging from around 13% to 80% for TRM and from 27% to 142% for FRP, contingent upon configuration. Al-Saidy et al. [25] conducted an experimental study on the flexural strengthening of reinforced concrete (RC) beams utilizing textile-reinforced mortars (TRMs), focusing specifically on the effects of bonding material, the number of textile layers, and anchorage design on strength improvement and ductility. Their results indicated that all reinforced beams obtained increased load capacity and enhanced deformation capacity relative to the reference specimen, which collapsed due to steel yielding followed by crushing of concrete. Elsanadedy et al. [11] examined the flexural strengthening of reinforced concrete (RC) beams utilizing textile-reinforced mortar (TRM), focusing on bond behavior, mortar composition, textile layers, and a comparison with CFRP. Their findings indicated that the use of TRM strengthening markedly improved flexural strength, stiffness, and load capacity, with strength increases ranging from roughly 39% to 91%, depending upon the mortar characteristics and the number of layers. Also, Yin et al. [26] investigated the influence of the total number of TRM layers (1, 2, and 3) and the condition of the textile surface on the flexural performance of reinforced concrete beams. The textile utilized was a hybrid, consisting of carbon fiber rovings oriented with the loading direction and glass fiber rovings oriented transversely. Their findings indicated that increasing the number of layers from 1 to 3 led to an improvement in flexural capacity ranging from 10% to 45%. Park et al. [27] carried out an experimental investigation on the flexural behavior of reinforced concrete (RC) beams strengthened with textile-reinforced mortar (TRM), emphasizing the efficacy of a locally produced alkali-resistant (AR) glass textile and the effects of TRM age, the quantity of textile layers, and the degree of pre-cracking. They found that TRM strengthening markedly improved cracking, yielding, and ultimate loads, with ultimate capacity augmentations of around 31%, 54%, and 72% for two, three, and four textile layers, respectively. Strength improvements increased roughly linearly with the number of layers.

On another point, some studies have addressed strengthening reinforced concrete beams using NSM technology. Wu et al. [28] studied the effect of the NSM method on the strengthening of reinforced concrete beams. The influence of several parameters, such as the number of NSM-CFRP bars, prestressing in NSM-CFRP, and the method of end treatment to mitigate end bond failure, on the performance of reinforced beams was examined. Their results indicated that the application of NSM-CFRP bars effectively enhances the flexural capacity of concrete beams. Sharaky et al. [29] conducted an experimental study on the flexural behavior of reinforced concrete beams enhanced with the near-surface-mounted (NSM) FRP method. The researchers confirmed improvements in yield load, which varied from around 124–156% for CFRP and 114–130% for GFRP, whereas ultimate load enhancements reached 155–166% and 141–159%, respectively. Ali et al. [30] examined the flexural strengthening of RC beams using an advanced NSM–glass fiber-reinforced polymer (GFRP) system, including a novel anchorage. The researchers noted that the hybrid anchorage system improved the maximum load capacity by approximately 2.6 times compared to the reference sample, as well as transferring failure to the GFRP rod and steel rupture, in addition to increasing stiffness and ductility.

Recent studies have emphasized the importance of interfacial bond behavior in composite cementitious systems. Bai et al. [31] showed that effective stress transfer across interfaces is critical to composite action. This is particularly relevant to externally bonded and near-surface-mounted strengthening systems, where debonding or bond-slip may limit the utilization of the strengthening material.

Although considerable research has investigated the flexural strengthening of reinforced concrete (RC) beams using CFRP, TRM, and NSM systems, most studies have examined these techniques independently or under different experimental conditions, limiting direct comparisons of their structural performance. Therefore, this study addresses this gap by experimentally comparing the flexural behavior of RC beams strengthened with CFRP sheets, carbon fiber plates, TRM systems—with carbon, basalt, and glass textiles—and NSM carbon and glass bars under identical testing conditions. The study provides a systematic evaluation of load capacity, stiffness, ductility, energy dissipation, and failure modes, offering practical guidance for selecting the most suitable strengthening technique according to the required structural performance.

## 2. Experimental Study

This experimental investigation examines the strengthening of reinforced concrete (RC) beams using different strengthening techniques. These include carbon fiber-reinforced polymer (CFRP) in two forms: sheets and plates, as well as textile-reinforced mortar (TRM) incorporating carbon, glass, and basalt textiles, in addition to the near-surface-mounted (NSM) technique. In this study, 22 reinforced concrete beams were cast, including one control beam. The remaining 21 specimens were strengthened with different strengthening techniques under a four-point bending test.

The key experimental parameters investigated in this study include the type of strengthening, textile type, number of textile layers, and the implementation of anchorage systems.

### 2.1. Test Specimens and Materials

**Figure 1:**
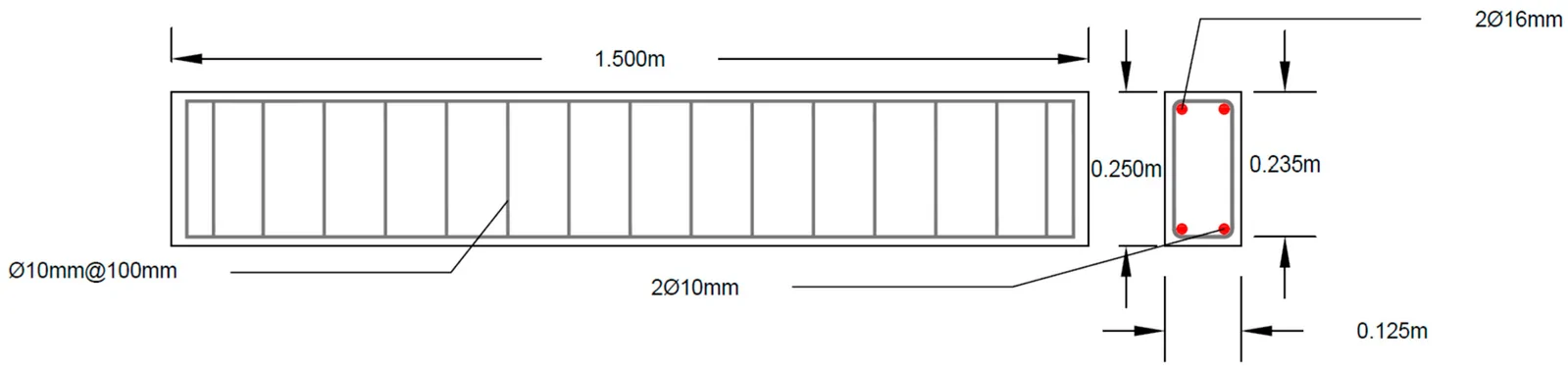
The details of RC beams.

The RC beam specimens were 1500 mm long and had a rectangular cross-section of 125 × 250 mm, as shown in Figure 1. Two sizes of reinforcing steel bars were used: 2Ø10 mm bars were placed in the tension zone, while 2Ø16 mm bars were used in the compression zone. Ø10 mm stirrups spaced at 100 mm were provided along the entire length of the specimens. The greater amount of top reinforcement was intentionally adopted to create a controlled flexural deficiency in the tension zone and to enable a clearer comparative evaluation of the strengthening techniques applied to this region. The lower tension reinforcement ratio promotes flexure-controlled behavior, whereas the higher compression reinforcement limits the possibility of premature failure in the compression zone. Accordingly, the expected response consists of flexural cracking, yielding of the tension reinforcement, and subsequent failure governed by the behavior of the applied strengthening system.

Two aggregate size ranges (0–4 mm and 4–11 mm) were utilized to make the concrete mixture with a target compressive strength of 25 MPa. The selected cement grade was CEM I 42.5 R. The water-to-cement ratio was set at 0.529. A plasticizer was added to enhance the workability of the concrete, and fly ash was incorporated as a mineral additive. The concrete consistency class was S3. The concrete mixture was prepared according to the mixing proportions specified for 1 m^3^ (Table 1) and subsequently brought to the laboratory by a concrete mixer truck. During the production of the test beams, five cubic specimens with dimensions of 150 × 150 × 150 mm were collected for every tested specimen. The collected specimens were examined on the day of the respective beam test. The results obtained from the cube samples were converted to the results obtained for the standard cylindrical sample (Ø150 × 300 mm^3^). 

Ribbed Ø16 steel bars were used for longitudinal reinforcement in the compression zone, and ribbed Ø10 bars were used for both stirrups and tension longitudinal reinforcement. The mechanical parameters of the reinforcing steel are presented in Table 2.

**Figure 2:**
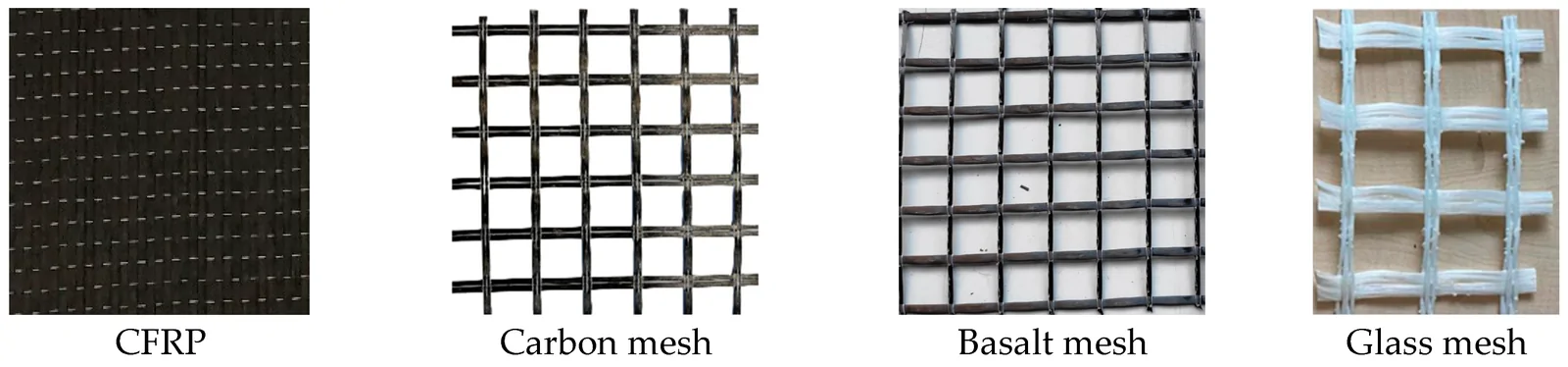
Textile types used in strengthening [33].

Four different fiber textiles were employed in the experimental study. The TRM strengthening technique employed carbon mesh-350, basalt mesh-350, and glass mesh, as shown in Figure 2. Carbon fiber-reinforced polymer (CFRP) was used as the strengthening technique with FRP sheets. Additionally, carbon fiber plates were used for the externally bonded strengthening system. The mechanical properties of FRP textiles are given in Table 3. These values were provided by the manufacturers.

For the TRM strengthening technique, a structural thick repair mortar, as detailed in Table 4, was selected instead of conventional plaster mixes to delay the delamination of the textiles from the concrete surface. The characteristics of the repair mortar were obtained from the manufacturer. The mortar was cement-based and single-component. In addition to the polymer ingredient, it also included fiber additives. Two-component epoxy was used in the process of installing CFRP sheets and carbon fiber plates. The mechanical properties of the epoxy are given in Table 4.

**Figure 3:**
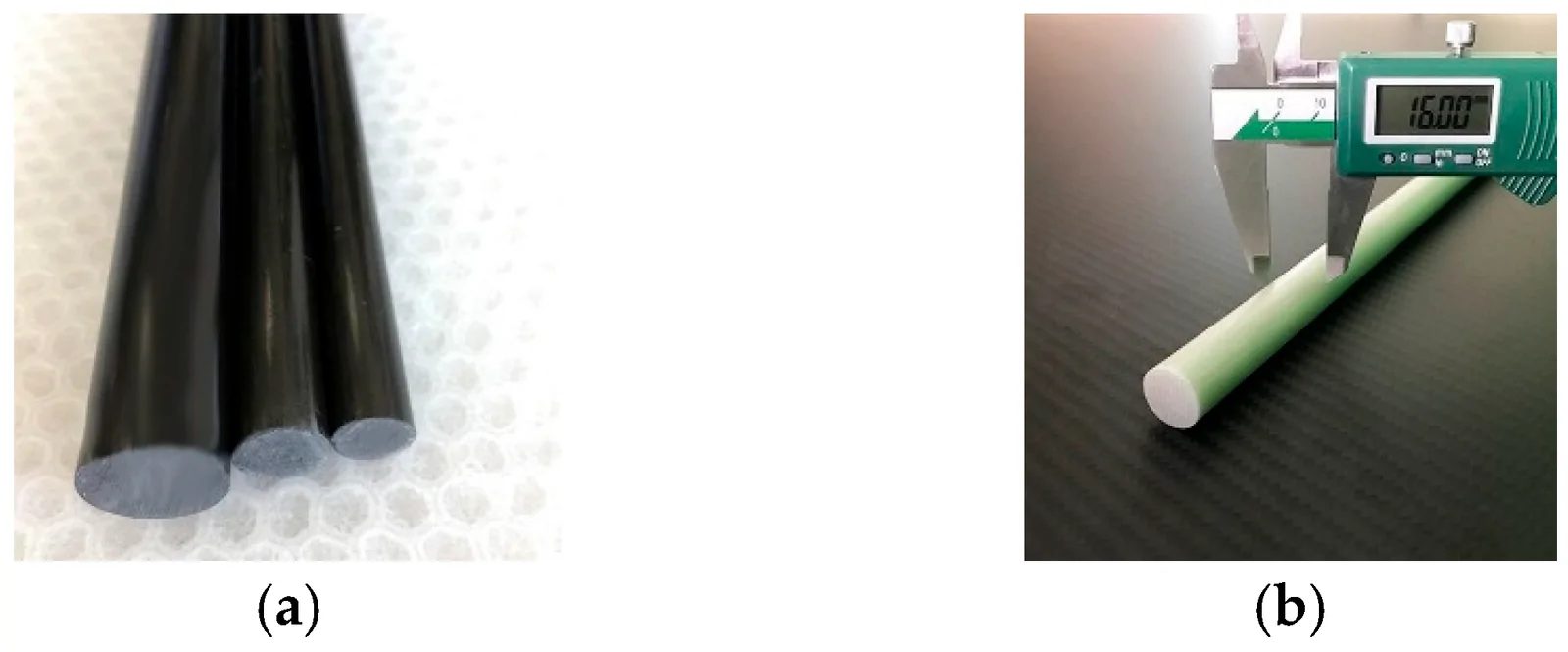
NSM bars: (**a**) carbon fiber bar; (**b**) glass fiber bar.

In the NSM strengthening technique, Ø16 mm carbon fiber and glass fiber rods were used, as shown in Figure 3. The mechanical properties of FRP bars are given in Table 5.

**Figure 4:**
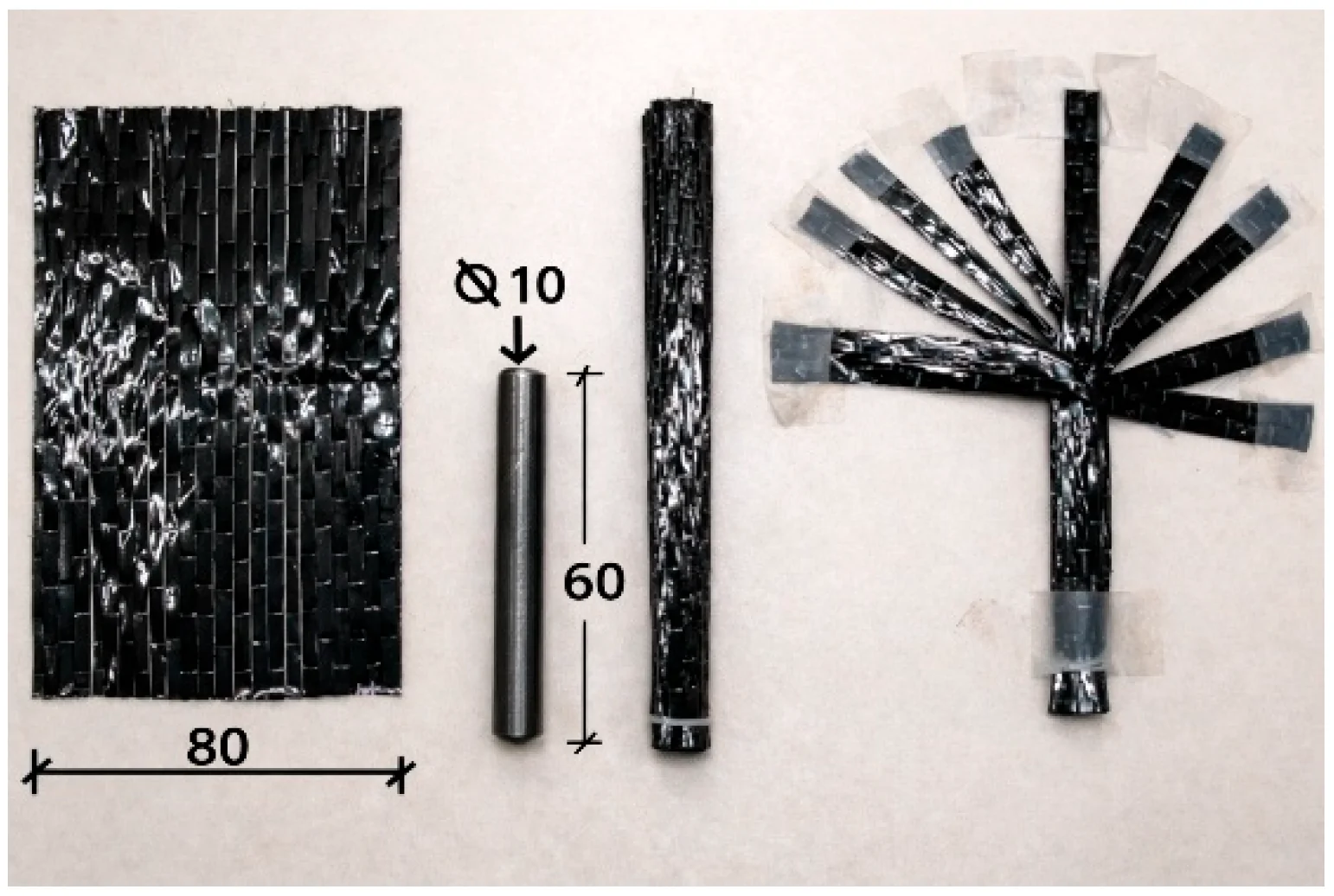
Details of the fan-type anchorage (dimensions in mm).

A fan-type anchorage system was used. For this, CFRP 300-C sheets and 10 mm diameter ribbed steel bars were used, as shown in Figure 4.

### 2.2. Strengthening Details of Specimens

**Figure 5:**
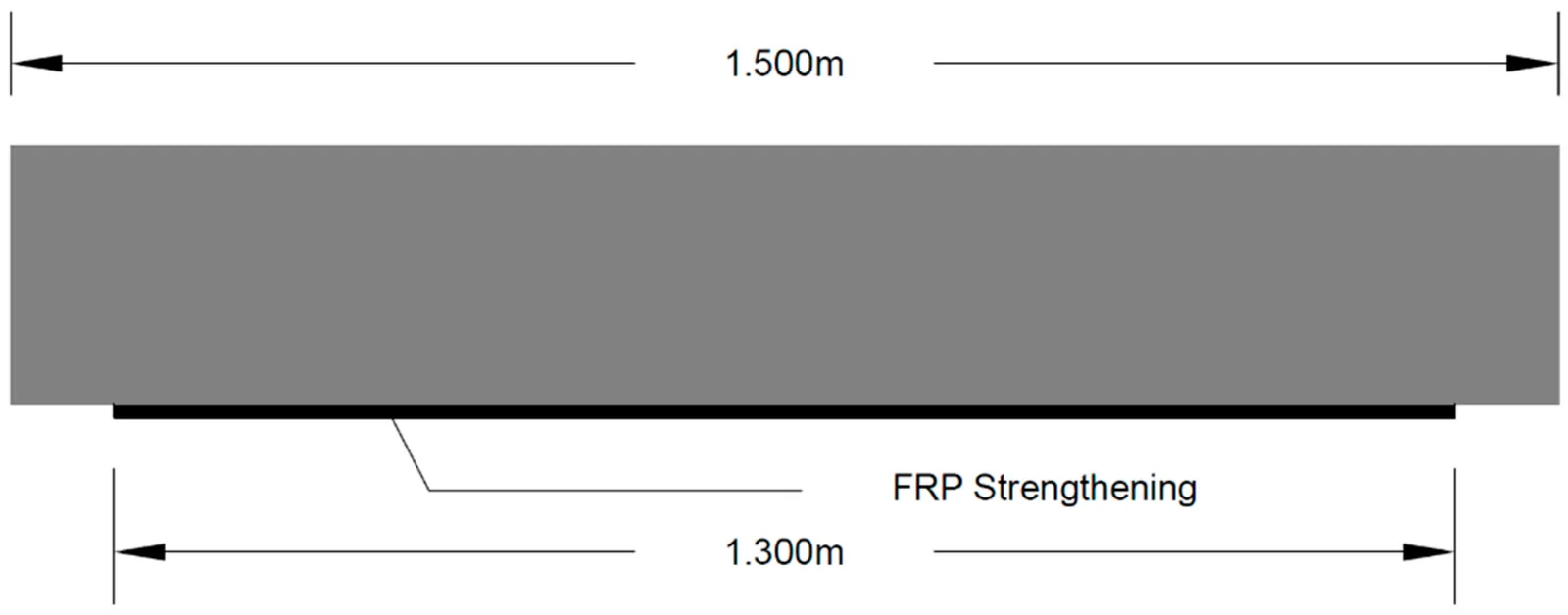
Strengthening scheme of RC beams.

**Figure 6:**
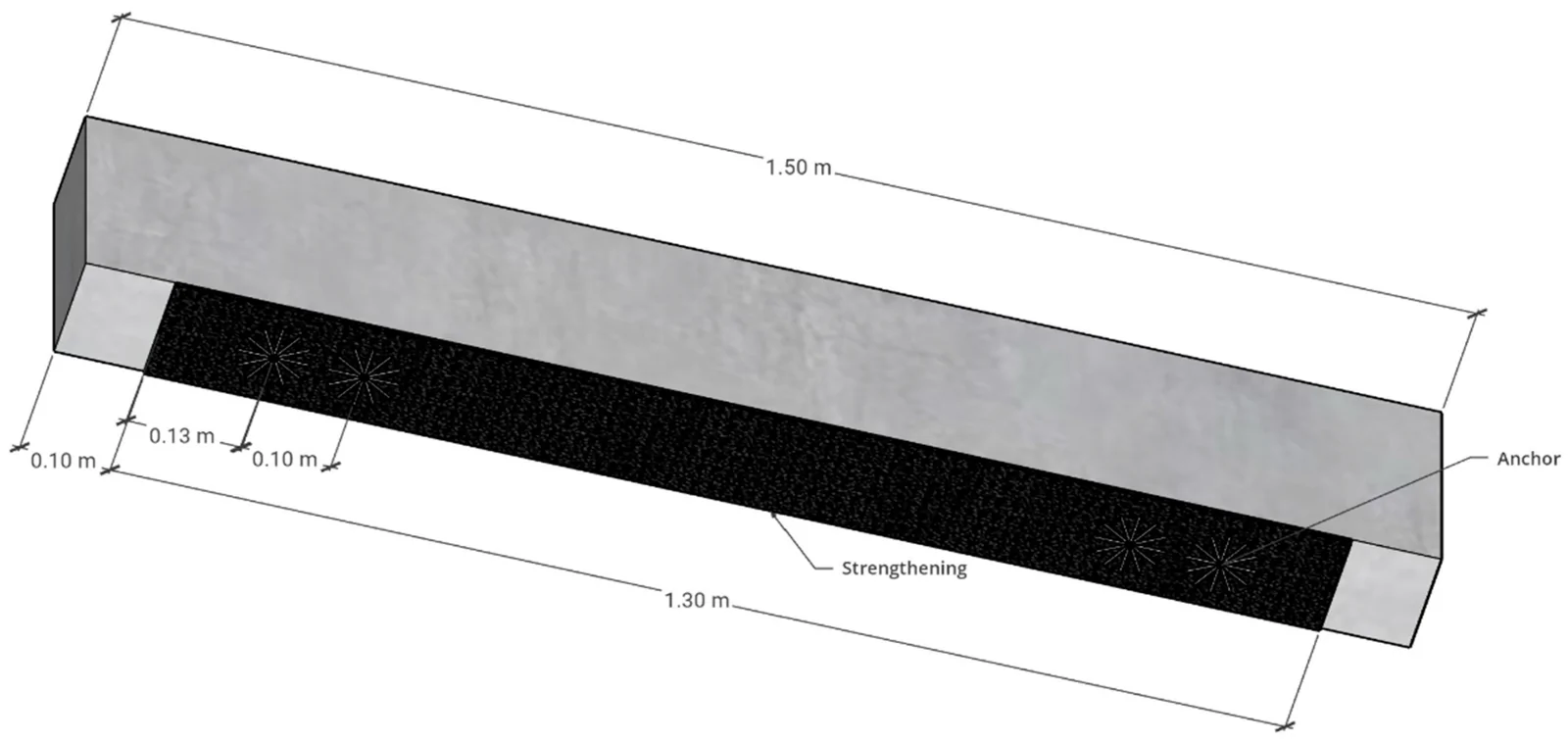
Anchor positions.

**Figure 7:**
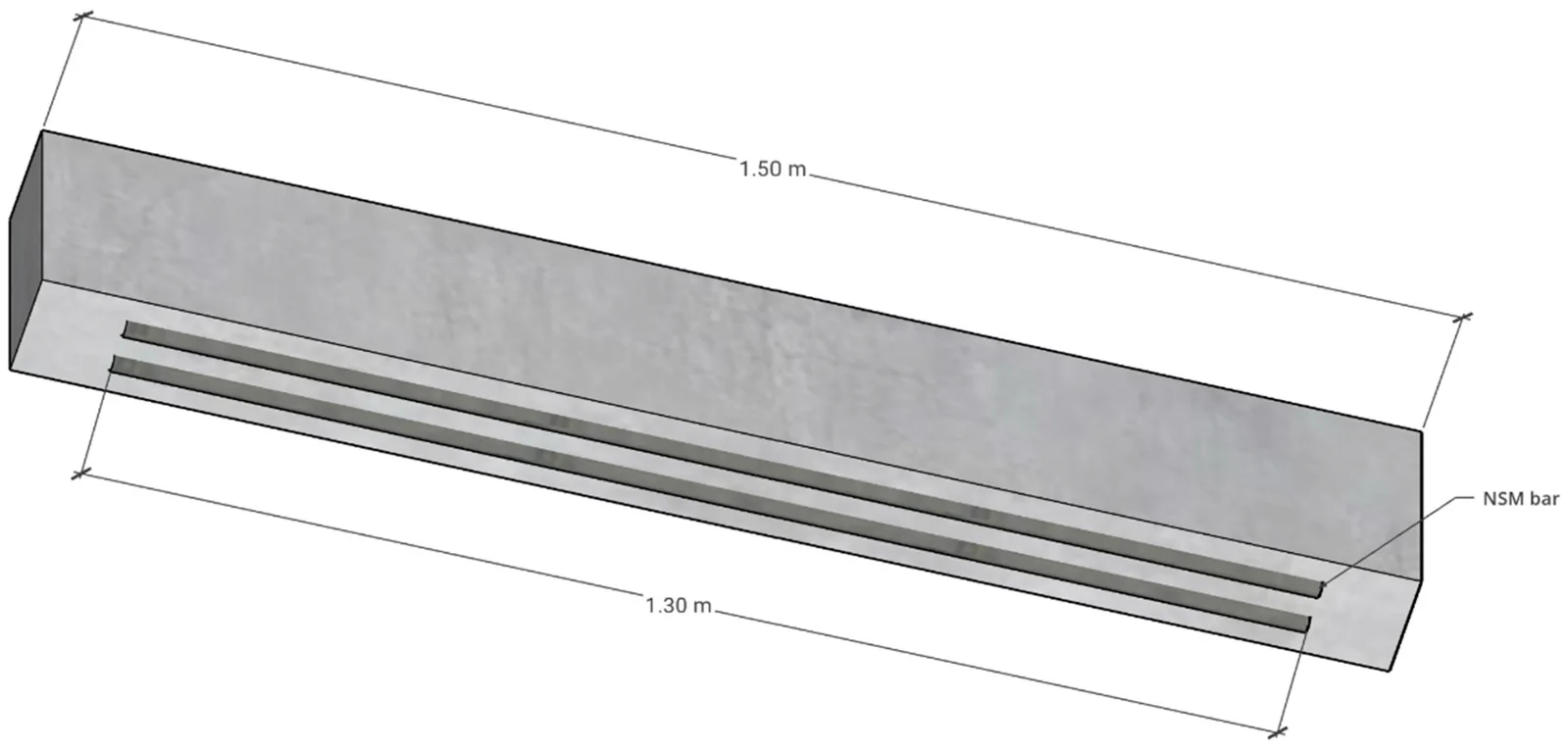
NSM bar scheme.

The specimens were divided into six groups according to the strengthening method. The first group consisted of four specimens strengthened using CFRP sheets with different numbers of layers and with or without anchors. The second group consisted of four specimens strengthened using carbon textile-reinforced mortar (CTRM) with different numbers of layers, with and without anchors. The third group consisted of four specimens strengthened using basalt textile-reinforced mortar (BTRM) with different numbers of layers and with or without anchors. The fourth group consisted of four specimens strengthened using glass textile-reinforced mortar (GTRM) with different numbers of layers and with or without anchors. The fifth group consisted of three specimens strengthened using carbon fiber plates with different numbers of layers and anchorage conditions. The sixth group consisted of two specimens strengthened using carbon and glass fiber NSM bars, and no additional end-anchorage system was included in either configuration. The use of anchorage was associated with the specific characteristics and expected failure mechanisms of the EBR strengthening system rather than being considered as a common variable for all strengthening configurations. Consequently, the NSM results presented in this study represent unanchored NSM configurations and permit comparison of the two bar materials under the same installation condition; the experimental program was not designed to evaluate the effect of end anchorage on NSM performance. In all strengthened specimens, the strengthening material was applied to the tension zone of the beam, as shown in Figure 5. For the reinforced concrete beams where an anchoring system was used, four fan-type anchors were installed within the tension zone. Two anchors were placed at each end of the beam, with a spacing of 10 cm between the two anchors at each end. The strengthening details of all groups, including specimen designation, type of strengthening, strengthening materials, number of layers, anchorage condition, and the concrete compressive strength of each test specimen, are summarized in Table 6. The positions of the anchors and NSM bars are shown in Figure 6 and Figure 7, respectively. 

### 2.3. Production and Strengthening of Specimens

**Figure 8:**
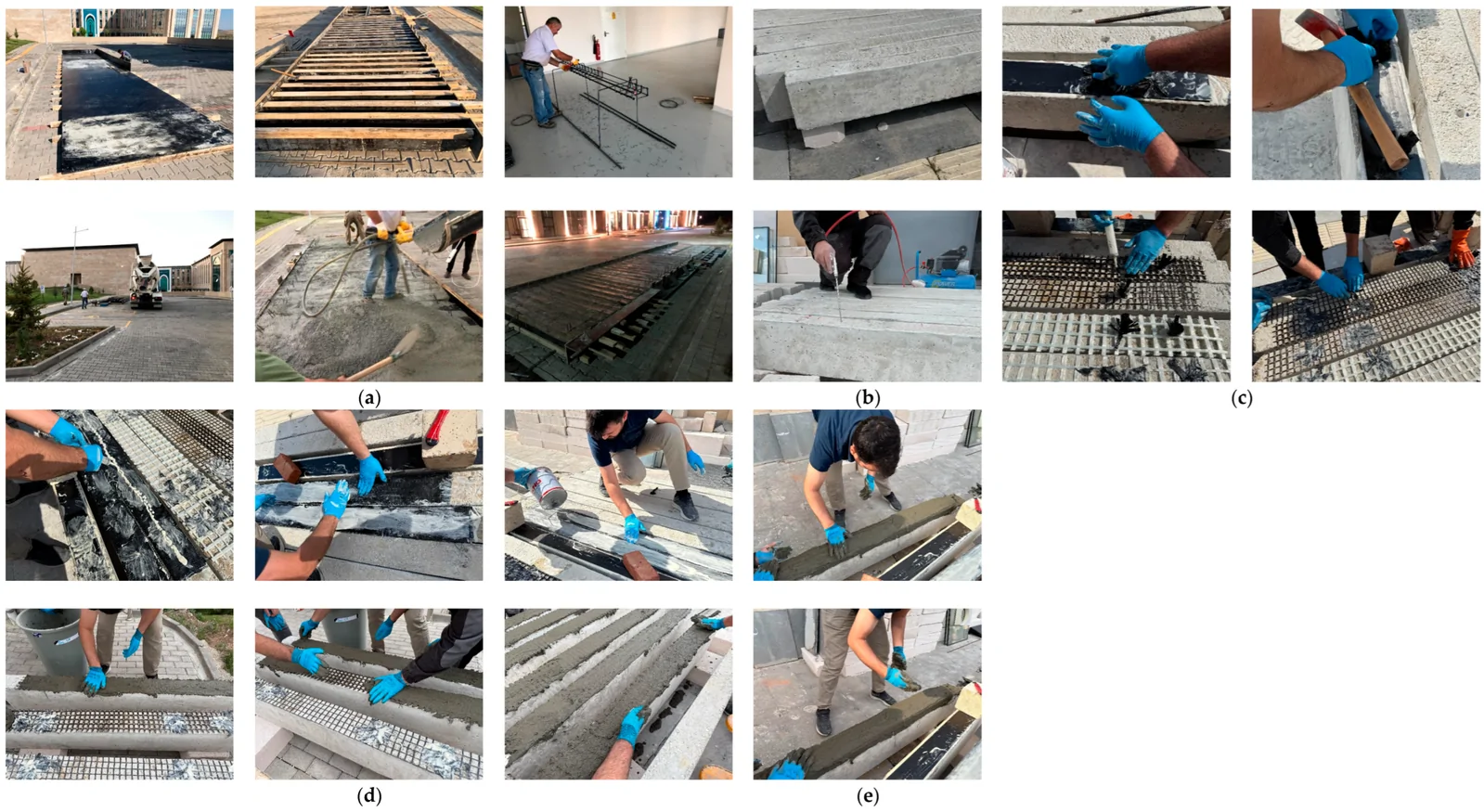
Production and strengthening procedures of the test specimens: (**a**) production of RC beams [33]; (**b**) concrete surface preparation; (**c**) fan-type anchor installation; (**d**) epoxy adhesive and repair mortar application; and (**e**) installation of NSM FRP bars.

Figure 8a illustrates the production stages of the RC test beams. The strengthening processes began after 28 days, following the completion of the curing process. First, the concrete surfaces were prepared, as shown in Figure 8b. In the subsequent step, the FRP textile was placed and fixed on the beam surface. For the specimens requiring anchorage, fan-type anchors were installed according to the strengthening configuration of each specimen. When fan-type anchors were used, a preliminary layer of epoxy was first applied to the anchorage region. The anchor was then inserted, and the FRP textile was fixed in place. The epoxy was allowed to cure for 24 h. The anchoring installation procedure is illustrated in Figure 8c.

In the final step, mortar was applied to the specimens strengthened using the TRM technique. Epoxy adhesive was applied to the specimens strengthened using the CFRP sheets and to those strengthened with carbon fiber plates, as shown in Figure 8d.

Figure 8e shows the installation process of glass and carbon bars for the specimens strengthened using the NSM technique.

### 2.4. Test Setup

The test specimens were subjected to a four-point bending load. The load was applied through a rigid steel loading frame using a hydraulic loading system. The beams had a total length of 1500 mm and a rectangular cross-section of 125 mm × 250 mm, with an effective depth (d) of 235 mm. The support span was 1400 mm, with each support positioned 50 mm from the corresponding beam end. The two concentrated loads were applied symmetrically, with a spacing of 570 mm between the loading points, thereby producing a 570 mm constant-moment region. Each loading point was located 465 mm from the corresponding beam end. Accordingly, the shear span, measured from the center of each support to the nearest loading point, was 415 mm. Based on an effective depth of 235 mm, the resulting shear-span-to-effective-depth ratio was (a/d = 415/235 = 1.77). The loading rate was maintained at 1 kN/s. The applied load and displacement measurements were recorded for all specimens.

**Figure 9:**
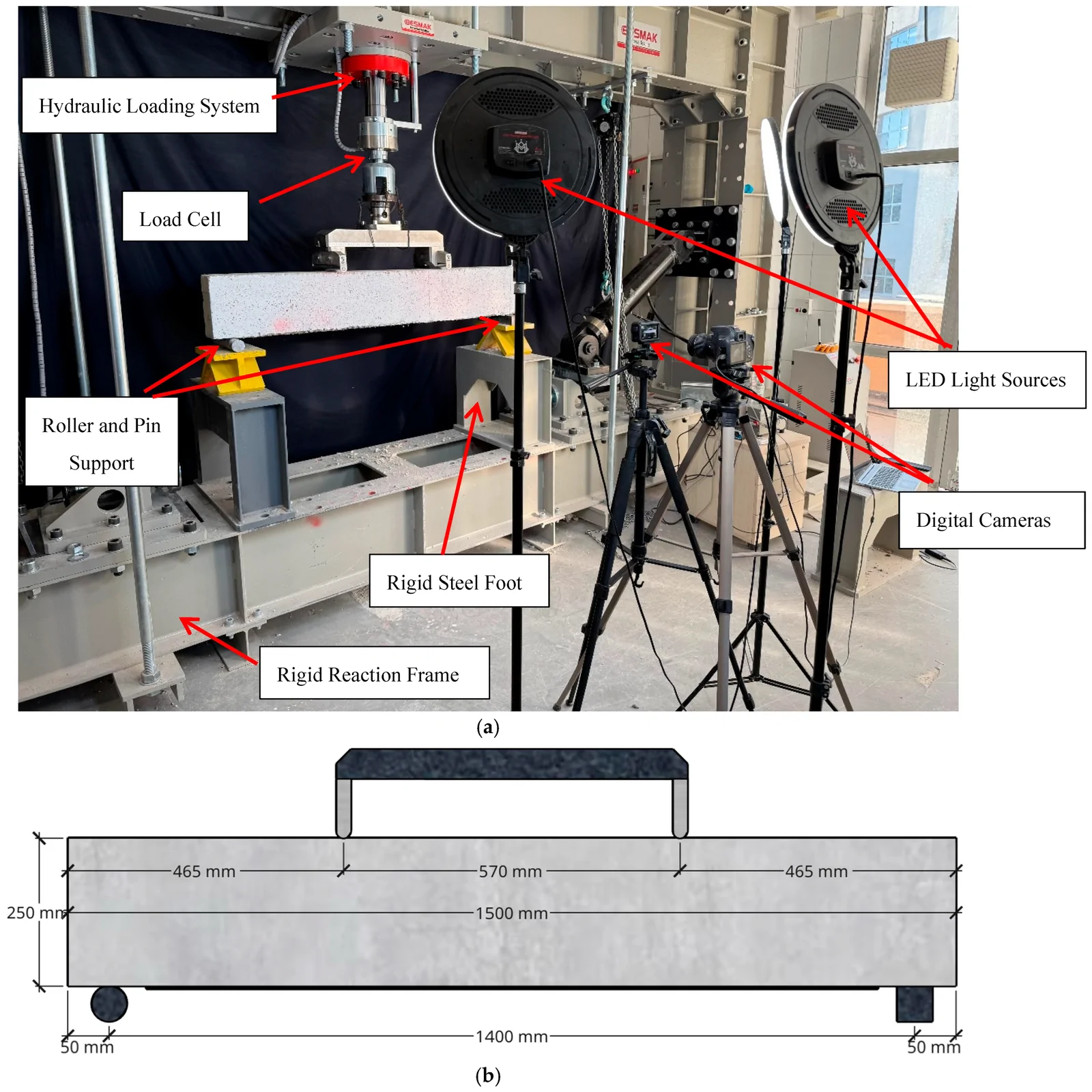
Test setup. (**a**) Actual experimental setup. (**b**) Dimensions and loading configuration.

Figure 9a shows the actual experimental setup, whereas Figure 9b presents the dimensions and loading configuration.

## 3. Experimental Results

**Figure 10:**
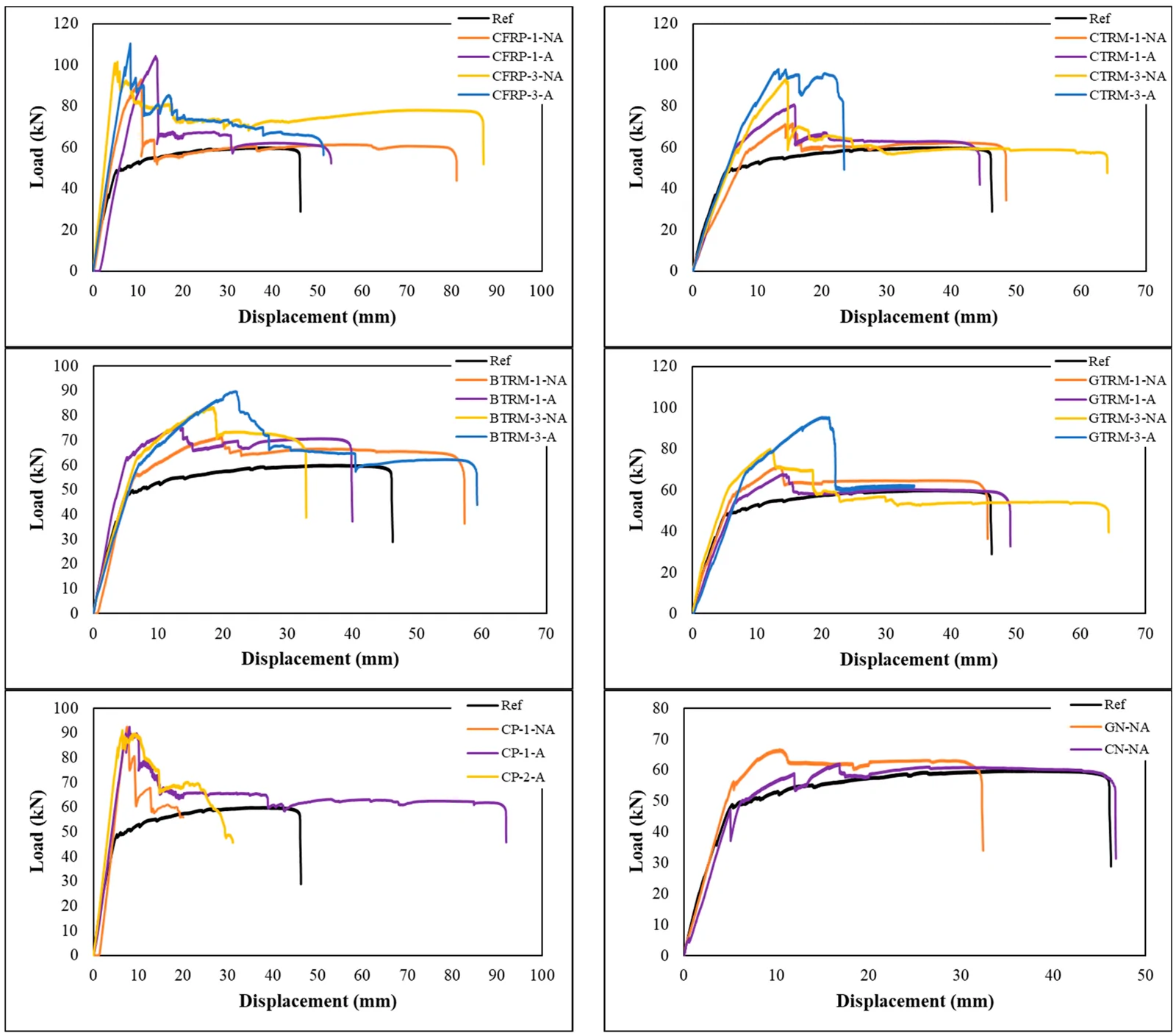
Load–displacement curves of test specimens.

The load–displacement data were collected from the experimental program and analysed based on the main investigated parameters. The load–displacement curves of the tested specimens are presented in Figure 10. In addition, the effects of these parameters were evaluated using several performance indicators, such as the ultimate load capacity, displacement at peak load, stiffness, ductility ratio, and energy dissipation capacity, through comparisons of the experimental results.

**Figure 11:**
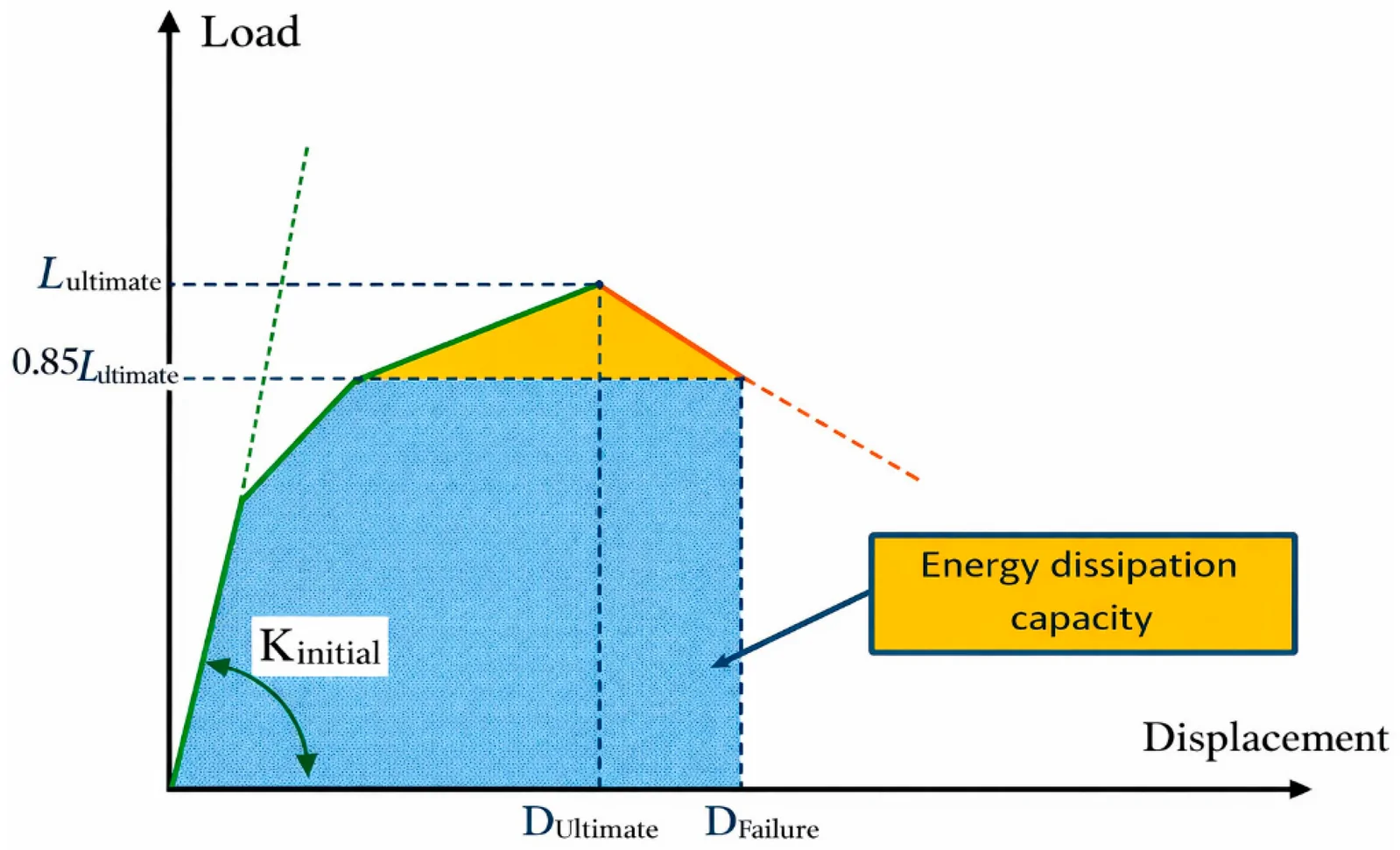
Systematic approach used for obtaining the test results of the specimens [33].

Figure 11 illustrates the procedure adopted to obtain the test results of the specimens. The ultimate load capacity was defined as the maximum load recorded on the load–displacement curves. For the displacement ductility calculation, the failure displacement (δ_0.85_) was defined as the post-peak displacement corresponding to 85% of the maximum load (0.85 P_max_) on the descending branch of the load–displacement curve. The peak-load displacement (δ_peak_) was defined as the displacement corresponding to P_max_. Accordingly, the displacement ductility ratio was calculated as μΔ = δ_0.85_/δ_peak_. In addition, the stiffness ratio was calculated from the load–displacement response. The energy dissipation capacity was determined as the area under the load–displacement curve up to the point of peak load.

Table 7 shows the maximum bearing capacity, displacement at ultimate load, stiffness, ductility ratio, and energy dissipation capacity.

The maximum load of the unstrengthened reinforced concrete beam under four-point bending was 60 kN at a displacement of 37 mm. The stiffness, ductility ratio, and energy dissipation capacity of the specimen were 1.59 kN/mm, 1.22, and 2487.45 kN-mm, respectively.

For the specimens of the first group, the ultimate load-carrying capacities of Specimen 2, Specimen 3, Specimen 4, and Specimen 5, as strengthened by the FRP wrap technique using carbon fiber-reinforced polymer (CFRP), increased by 54.94%, 73.80%, 69.17% and 84.04%, respectively, reaching 92.98 kN, 104.3 kN, 101.52 kN and 110.44 kN. The stiffness and ductility ratio of Specimen 2 were 8.69 kN/mm and 1.01, respectively, while its energy dissipation capacity increased by 93.62%, reaching 4816.25 kN-mm. For Specimen 3, the stiffness and ductility ratio were 7.49 kN/mm and 1.03, respectively, and the energy dissipation capacity increased by approximately 32%, reaching 3279.86 kN-mm. In Specimen 4, the stiffness and ductility ratio were 18.83 kN/mm and 1.56, respectively, while the energy dissipation capacity reached 6509.34 kN-mm, representing an increase of 161.69%. For Specimen 5, the stiffness and ductility ratio were 13.32 kN/mm and 1.01, respectively, and the energy dissipation capacity increased by 45%, reaching 3609.37 kN-mm.

As for the specimens of the second group, the maximum load capacity of Specimens 6, 7, 8, and 9, after being strengthened with a carbon textile mesh (type 350) using the TRM strengthening method, increased by 19.41%, 34.74%, 55.26%, and 63.32%, respectively, reaching 71.66 kN, 80.86 kN, 93.17 kN, and 98.01 kN. The stiffness and ductility ratio in Specimen 6 were 4.64 kN/mm and 1.08, respectively, and its energy dissipation capacity increased slightly by 10%, reaching 2738.11 kN-mm. For Specimen 7, the stiffness was 5.17 kN/mm, and the ductility ratio was 1.2, and the energy dissipation capacity increased by about 7.06%, reaching 2663.08 kN-mm. The stiffness and ductility ratio of Specimen 8 were 6.47 kN/mm and 1.02, respectively. The energy dissipation capacity reached 3795.81 kN-mm, representing an increase of 53%. In Specimen 9, the stiffness and ductility ratio were 7.4 kN/mm and 1.73, respectively. In this specimen, a decrease in energy dissipation capacity was observed, reaching 1692.89 kN-mm.

The specimens of the third group, which included Specimens 10, 11, 12, and 13, were strengthened using a basalt textile mesh (type M350) with the TRM technique. The increases in the ultimate load capacity were 18.85%, 26.60%, 38.89%, and 49.68%, respectively. The stiffness values of the specimens in this group were 3.62 kN/mm, 5.75 kN/mm, 4.51 kN/mm, and 4.09 kN/mm, respectively. The corresponding ductility ratios were 2.88, 3.01, 1.02, and 1.14, respectively. On the other hand, the energy dissipation capacities were 3493.4 kN-mm, 2590.99 kN-mm, 2145.35 kN-mm, and 3809.5 kN-mm, respectively.

An increase in the ultimate load capacity was recorded for the specimens of the fourth group, which included Specimens 14, 15, 16, and 17, strengthened using glass textile mesh with the TRM technique. The increases were 19.16%, 12.98%, 33.21%, and 58.99%, respectively, reaching 71.51 kN, 67.8 kN, 79.94 kN, and 95.41 kN. For Specimen 14, the stiffness and ductility ratios were 5.39 kN/mm and 3.39, respectively. The energy dissipation capacity showed a slight increase of approximately 9.5%. Specimen 15 recorded a stiffness of 4.86 kN/mm and a ductility ratio of 3.43, with a modest increase in energy dissipation capacity of about 10%. For Specimen 16, the stiffness and ductility ratios were 6.67 kN/mm and 1.55, respectively, and the energy dissipation capacity increased by approximately 45%. In Specimen 17, a significant decrease in energy dissipation capacity was observed, reaching 2188 kN-mm, where the stiffness and ductility ratios were 4.75 kN/mm and 1.09, respectively.

The specimens that were strengthened using carbon fiber plates, which include specimens of the fifth group of 18, 19 and 20, recorded an increase in the maximum load capacity of 54.04%, 54.07% and 51.8%, respectively, amounting to 92.44 kN, 92.46 kN and 91.12 kN. The stiffness values of the specimens in this group were 12.42 kN/mm, 11.51 kN/mm, and 14.13 kN/mm, respectively, while the ductility ratios were 1.06, 1.26, and 1.97, respectively. Specimens 18 and 20 showed a decrease in energy dissipation capacity, while Specimen 19 showed an increase in energy dissipation capacity of 133%.

The sixth group consisted of NSM-strengthened specimens. The increases in the ultimate load capacity of Specimens 21 and 22 after strengthening using the NSM technique were slight, and the increases reached only 11.20% and 3.52%, respectively. The stiffness values of the specimens in this group were 6.46 kN/mm and 3.69 kN/mm, respectively, while the ductility ratios were 3.11 and 2.77, respectively.

## 4. Discussion

### 4.1. Failure Modes

**Figure 12:**
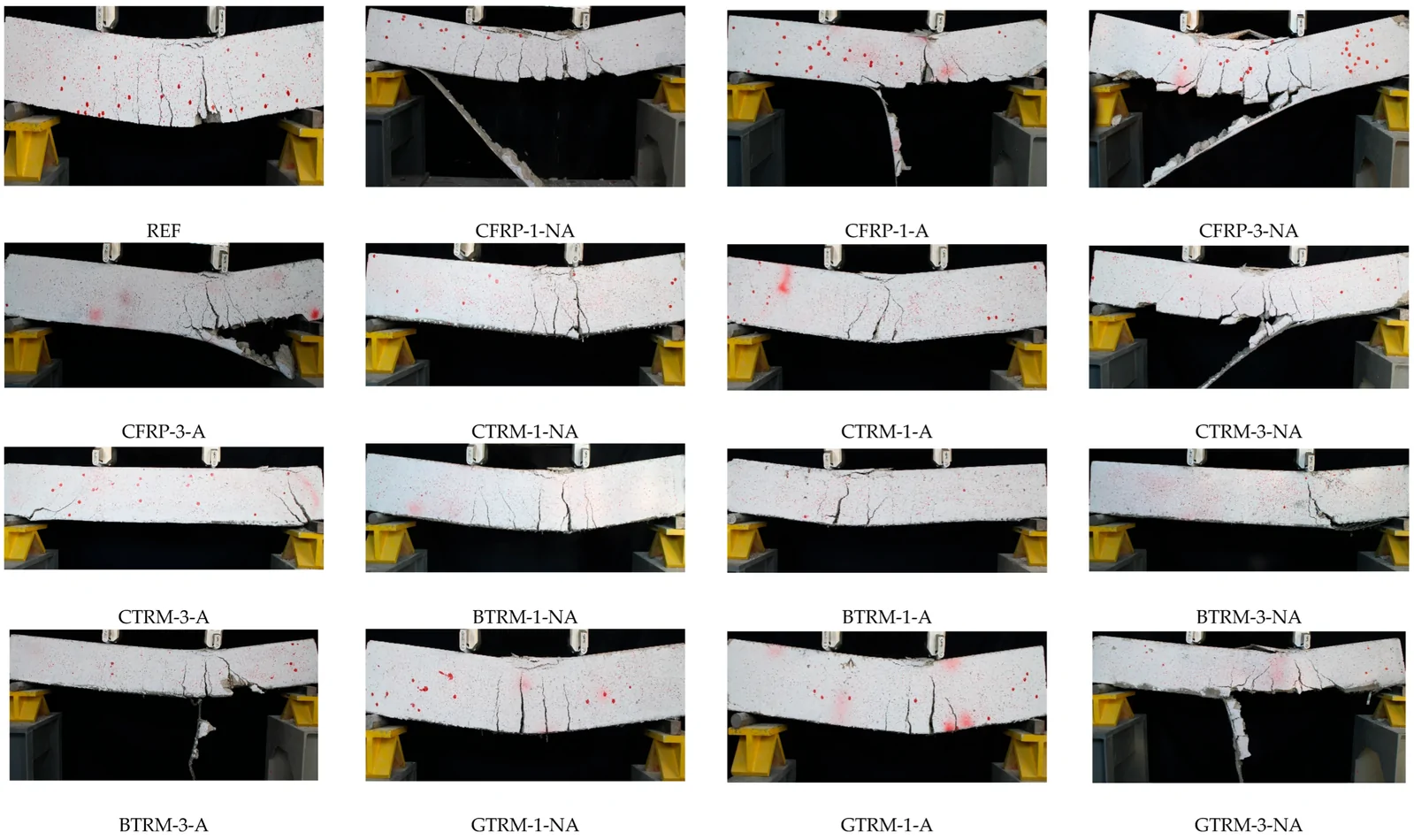

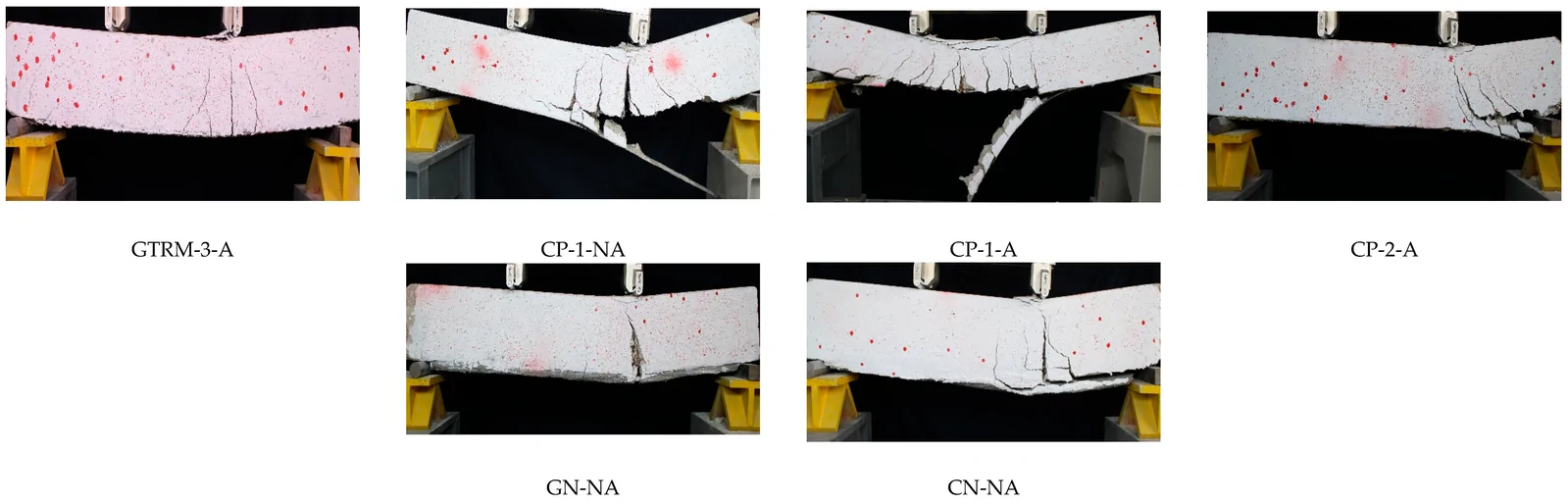
Failure modes and damage distribution of the specimens.

Figure 12 presents the failure modes of all tested specimens. The failure of the reference beam started with cracking in the tension zone of the beam, and as the load increased, the cracks developed and disseminated until the concrete crushing occurred at the compression zone. At this stage, the reinforcing bars had yielded.

Specimen CFRP-1-NA showed a flexural failure resulting from premature CFRP debonding with separation of the concrete cover. Multiple flexural cracks developed in the constant moment region. The spread of the cracks was followed by an interfacial/bond crack, which led to the debonding of the CFRP strip from the concrete cover. This led to concrete crushing in the tension zone and yielding of the reinforcing steel. Localized concrete crushing/spalling was also observed in the compression zone near the loading area during the ultimate stage.

Specimen CFRP-1-A with fan-type anchorage exhibited a flexural failure mode. Extensive flexural cracks developed in the tension zone, followed by crushing/spalling of the concrete in the compression zone. In contrast to the CFRP-1-NA specimen, in which no fan-type anchor was used, no clear premature delamination of the CFRP textile was observed, indicating the effectiveness of the fan-type anchor in improving force transfer and delaying failure. At ultimate load, failure was accompanied by cracking and separation of the bottom concrete cover, leading to the rupture of the CFRP textile with a significant portion of the concrete separating in the tension zone.

Specimen CFRP-3-NA failed primarily due to premature debonding associated with the separation of the concrete cover in the tension zone. Multiple flexural cracks formed within the tension zone, and as the load increased, a longitudinal separation crack propagated along the bottom of the concrete cover. This caused a portion of the concrete cover to detach along with the bonded CFRP laminate. Concrete crushing was also observed in the compression zone at the ultimate load.

Specimen CFRP-3-A exhibited predominantly cover separation and anchor rupture. Unlike the unanchored CFRP-3-NA specimen, no premature fiber separation was observed in the CFRP. The fan-type anchors enhanced force transfer and delayed interfacial separation, thereby allowing for greater utilization of the CFRP strengthening system. However, this caused the weak point to shift to the concrete substrate, resulting in severe delamination of the concrete cover and the collapse of the bottom portion of the concrete bonded to the carbon fibers, as well as damage to the anchor. Consequently, the prevailing failure can be classified as an anchorage-assisted flexural failure with concrete cover separation.

Specimen CTRM-1-NA showed a primarily flexural response characterized by multiple vertical cracks across the constant moment zone. At the ultimate load, localized crushing of the concrete was observed in the compression zone below the loading point. In the tension zone, the TRM strengthening layer exhibited cracks and localized delamination with exposed carbon textiles protruding, indicating slippage and pulling of the fabric from the mortar matrix. The failure mode can be classified as a failure in the TRM bond caused by bending, involving cracking in the mortar, localized delamination, and textile slippage/pull-out.

Specimen CTRM-1-A exhibited a failure pattern very similar to that of the CTRM-1-NA specimen. In this specimen, the TRM layer was largely attached to the concrete surface, indicating that the anchors were effective in delaying widespread debonding. The presence of localized cracks in the mortar matrix and the exposure of textile fibers near the main flexural cracks suggest that failure involved progressive TRM bond deterioration and textile pull-out rather than full textile rupture. Therefore, the failure can be described as a flexure-induced TRM bond failure with localized debonding and textile slippage.

Specimen CTRM-3-NA exhibited a bending response with some vertical cracks. As the load increased, delamination occurred along the tension face, resulting in the separation of a large portion of the carbon TRM strip along with a portion of the concrete cover. Therefore, the failure can be described as debonding of the TRM matrix with separation of a portion of the concrete cover.

In Specimen CTRM-3-A, the failure behavior was different, with failure concentrated primarily in the end regions rather than at midspan. The use of anchors had a very effective impact. The failure mode in the CTRM-3-A specimen was flexural-shear cracking in the end zone and debonding of the TRM.

Specimen BTRM-1-NA exhibited a ductile flexural failure. It was characterized by progressive matrix cracking, basalt textile rupture, and crushing of the concrete in the compression zone. Widespread vertical bending cracks can also be observed across the region of constant moment, indicating excellent stress redistribution. A clear rupture in the basalt fibers is visible within the widening initial cracks, indicating the tensile efficiency of the strengthening material.

Specimen BTRM-1-A exhibited a flexural failure mode, with the development of progressive cracking in the mortar matrix and tensile rupture of the basalt textile, culminating in concurrent concrete crushing in the compression zone. Extensive principal flexural cracks can be observed across the constant moment zone, indicating excellent stress redistribution. The anchors successfully maintained the composite action throughout the loading, thereby significantly preventing premature detachment of the concrete cover or interfacial debonding.

Specimen BTRM-3-NA showed mainly flexural behavior. The failure occurred near the right end, rather than, as is usually the case, midspan. As the load increased, severe cracks and concrete damage appeared near the support due to high bond and shear stresses. Mortar rupture and pulling of the basalt fibers were also observed, indicating localized detachment and slippage within the fabric, with the possibility of partial yarn rupture. The failure can be classified as a flexural-shear failure.

Specimen BTRM-3-A exhibited a sudden and brittle failure mode, with the anchors playing a key role in stabilizing the TRM until a brittle detachment occurred in the concrete cover as a result of tension. A longitudinal strip of the bottom concrete cover detached along with the TRM, exposing the internal reinforcing steel, indicating that the strengthening system developed high bond forces.

The unanchored Specimen GTRM-1-NA exhibited an ideal, ductile flexural failure, characterized by vertical cracks in the constant moment zone, glass textile rupture, and crushing of the concrete.

Specimen GTRM-1-A showed a flexural TRM bond failure with localized textile rupture. Vertical flexural cracks can be observed in the constant moment zone, in addition to cracking of the mortar matrix accompanied by ultimate tensile rupture of the glass textile. The top surface also shows concrete crushing in the compression zone beneath the loading point.

Specimen GTRM-3-NA exhibited a brittle flexural debonding failure with concrete cover separation in the tension zone. Multiple flexural cracks formed in the zone of constant moment. At ultimate load, a large strip of the bottom concrete cover detached together with the glass TRM layer, leaving the reinforcing bars uncovered. This indicates that, with three layers of TRM and no anchors, it was not feasible to effectively transfer the load across the interface between the mortar–textile–concrete interface. Consequently, the failure behavior was substrate/cover breakout and debonding.

Specimen GTRM-3-A exhibited a predominantly flexural TRM bond failure with localized textile slippage and rupture, instead of the severe delamination in the concrete cover observed in GTRM-3-NA.

Specimens CP-1-NA and CP-1-A both exhibited a premature flexural debonding failure with concrete cover separation of the carbon plate-strengthened tension face. Also, it can be observed that the carbon fiber plate detached completely along the span, resulting in a large section of the concrete cover being torn away and fully exposing the internal rebars.

Specimen CP-2-A showed an end-zone flexural-shear failure. The carbon fiber plate remained attached due to the use of anchors, but severe cracking and concrete spalling developed near the support area. The anchors delayed plate debonding, resulting in the damage being transferred from the bond interface to the concrete substrate.

Specimen GN-NA exhibited a flexural failure governed entirely by severe bond-slip (pull-out) of the strengthening bars. A wide flexural crack can be seen in the constant moment region, with the two glass bars bridging the gap remaining completely intact, indicating that the composite has lost its action.

Specimen CN-NA failed prematurely due to severe bond-slip. High shear stresses overwhelmed the mortar’s bond capacity, causing the solid carbon bars to pull out freely. This resulted in a single, massive flexural crack and a complete loss of composite action, leaving the carbon bars completely intact and their high tensile strength unutilized.

In the TRM specimens, matrix cracking represented an important stage in the development of composite action rather than an ultimate failure mechanism by itself. Following the cracking of the cementitious matrix, the tensile forces were gradually transferred to the textile strengthening via the bond between the textile–matrix bond and mechanical interlock. When several cracks gradually appeared in the matrix, particularly those observed in the single-layer BTRM specimens, the textiles acted to bridge these cracks and promote stress redistribution before textile rupture and concrete crushing. Conversely, when the tensile force developed by the textile exceeded the available textile–matrix, matrix–substrate, or concrete–cover resistance, the damage became localized, and the response shifted toward textile slippage, TRM debonding, or concrete–cover separation. This transition was particularly noticeable in the three-layer specimens. BTRM-3-A exhibited sudden concrete–cover detachment together with the TRM layer, while GTRM-3-NA showed brittle debonding and substrate/cover separation. Thus, increasing textile strengthening did not necessarily produce a more ductile response because the larger tensile force developed by the multilayer system increased the demand on the bond interfaces and concrete substrate. Once these transfer mechanisms became governing, failure occurred before full exploitation of the textile tensile capacity.

### 4.2. Effect of the Strengthening Type

The type of strengthening material had a direct and significant effect on the structural behavior of tested RC beams, especially in terms of ultimate load capacity, stiffness, ductility, and energy dissipation. Compared with the reference specimen, all strengthening systems significantly improved flexural performance, with the degree of improvement varying depending on the type of strengthening.

Among the investigated strengthening systems, the CFRP specimens exhibited ultimate-load increases of 54.94–84.04% relative to the reference specimen. The maximum ultimate load for the CFRP-3-A specimen was recorded at 110.44 kN, representing an increase of 84.04% compared with the reference specimen’s value of 60.01 kN. CFRP-3-NA recorded the highest stiffness value compared with the other specimens, at 18.83 kN/mm, and compared with the reference specimen’s value of 1.59 kN/mm.

The effect of strengthening using carbon plates (CPs) is also evident, where the ultimate loads reached 91.12–92.46 kN, corresponding to increases of approximately 51.80–54.07% relative to the reference beam. Their stiffness ranged from 11.51 to 14.13 kN/mm, while considering their more sensitive behavior in terms of bond conditions and anchorage effectiveness. The use of carbon textile-reinforced mortar (CTRM) also provided a marked improvement, exhibiting ultimate-load increases ranging from 19.41% to 63.32%, though it was less effective than the CFRP systems. In contrast, the TRM systems reinforced with basalt and glass textiles, as well as the NSM bar system, showed a different structural contribution. However, the use of these techniques resulted in a lower increase in ultimate load capacity compared to CFRP and carbon plates. The relatively small increase in ultimate load capacity of NSM specimens can be explained by the fact that in both NSM specimens, the strengthening bars remained essentially intact while substantial slip occurred along the bar–bonding material–concrete groove interface. In GN-NA, the glass FRP bars bridged the principal flexural crack but subsequently experienced severe bond-slip/pull-out. Similarly, CN-NA failed through pronounced pull-out of the carbon FRP bars without rupturing the bars themselves. Therefore, the available tensile capacity of the NSM strengthening was not fully mobilized before loss of composite action. The results indicate that the performance of the investigated NSM configurations was bond-controlled rather than strengthening-strength-controlled. Once the bond resistance along the groove was exceeded, additional tensile capacity of the FRP bars could not be effectively transferred to the surrounding concrete, limiting the contribution of the strengthening system to the flexural capacity. The deformation response depended strongly on the strengthening configuration. The one-layer BTRM specimens exhibited ductility ratios of 2.88 and 3.01, while GTRM-1-NA and GTRM-1-A reached 3.39 and 3.43, respectively, compared with 1.22 for the reference beam. The NSM specimens exhibited ratios of 3.11 and 2.77, indicating a less brittle response and a high ability to withstand deflection after cracking. In turn, the NSM specimens exhibited a slight improvement in ultimate load-bearing capacity, but relatively high ductility. However, several of the three-layer TRM specimens exhibited ductility ratios close to, or lower than, that of the reference specimen, indicating that the increase in the amount of strengthening did not systematically improve the deformation capacity. These results indicate that the strengthening method used not only affects the degree of improvement in strength but also influences the failure characteristics of the beam. Therefore, CFRP and carbon plate systems may be better when the main goal is to increase strength and stiffness. On the other hand, TRM and NSM systems may be preferable when enhanced deformability and a more gradual failure response are required.

### 4.3. Effect of Number of Layers

An increase in the number of strengthening layers leads to an increase in the axial stiffness of external strengthening. Provided that there is sufficient bond between the strengthening and the substrate, this, in turn, improves the strengthening’s capacity to resist and transfer tensile forces after cracking. The present CFRP results illustrate both the benefit and the bond limitation of this mechanism. Increasing the number of strengthening layers in the CFRP from one to three layers led to an increase in the ultimate load capacity of 9.2% without anchors and 5.9% with anchors, while the increase in stiffness was 116.7% and 77.8%, respectively. This indicates that the relatively modest increase in strength achieved by increasing the number of strengthening layers did not translate proportionally into bending capacity. The failure observations provide a mechanical explanation for this behavior. CFRP-3-NA developed extensive concrete cover separation and premature debonding, whereas CFRP-3-A eventually developed cover separation and anchor damage. As the axial capacity of external strengthening has increased, so too has the need for a load-transfer mechanism between the concrete and the CFRP, and the concrete substrate has become more important in controlling the ultimate response.

A similar mechanism was observed in the TRM groups. Increasing from one to three textile layers raised ultimate load by 21.2–30.0% for CTRM, 16.9–18.2% for BTRM, and 11.8–40.7% for GTRM, but the BTRM and GTRM ductility ratios decreased by approximately 54–69% in the corresponding layer comparisons. Also, it was observed that the energy dissipation of CTRM-3-A was 1692.89 kN-mm compared with 3795.81 kN-mm for CTRM-3-NA, corresponding to a reduction of approximately 55.4%. Similarly, GTRM-3-A dissipated 2188.12 kN-mm compared with 3609.53 kN-mm for GTRM-3-NA, representing a reduction of approximately 39.4%. This behavior indicates that increasing the number of strengthening textiles together with anchorage enhanced force transfer and peak-load resistance but did not necessarily improve the beam’s ability to sustain deformation while carrying load. For CTRM-3-A, the observed damage shifted toward localized flexural-shear cracking in the end zone and the debonding of the TRM, while GTRM-3-A exhibited a predominantly flexural TRM bond failure with localized textile slippage and rupture. These more localized failure mechanisms limited deformation and post-cracking load redistribution compared with the corresponding unanchored three-layer specimens, thereby reducing the area under the load–displacement response and consequently the measured energy dissipation. The single-layer BTRM and GTRM specimens generally exhibited distributed matrix cracking and, in several cases, textile rupture, whereas the three-layer specimens more often developed localized debonding, concrete–cover separation, or end-zone damage. This indicates that the additional textile initially improved crack resistance and tensile strength, but at the same time, the higher composite rigidity led to an increase in the force that had to be transferred through the cracked matrix and the interface between the TRM and concrete. Once bond or substrate resistance governed, further textiles could not be fully utilized, and the response became more localized.

Regarding the carbon-plate specimens, increasing the number of plate layers in the anchored specimens from one layer (CP-1-A) to two layers (CP-2-A) increased the stiffness from 11.51 to 14.13 kN/mm, while slightly reducing the ultimate load from 92.46 to 91.12 kN. In contrast, the maximum recorded displacement and energy dissipation decreased by 66.2% and 64.8%, respectively. Specimen CP-2-A exhibited end-zone flexural–shear failure while the carbon plate remained attached. These observations indicate that the additional plate layer enhanced the elastic and post-cracking restraint; however, it did not improve the peak load resistance, as the governing demand shifted toward the plate-end/anchorage region and the concrete substrate. Therefore, the effect of the number of plate layers should be evaluated in conjunction with the bond-transfer capacity and the associated failure mode.

### 4.4. Effect of Anchorage

For the CFRP specimens, the use of anchors resulted in an increase in the ultimate load capacity of 12.2% for the single-layer specimen and 8.8% for the three-layer specimen. More importantly, the failure mode associated with these specimens changed from early premature failure of the CFRP layer (debonding/cover separation in CFRP-1-NA and CFRP-3-NA) to delayed delamination accompanied by CFRP rupture in CFRP-1-A and cover separation, as well as rupturing of the anchor in CFRP-3-A. The DIC maps also show that the anchored specimens retained pronounced strain localization near the final damage region. Overall, these observations indicate that the use of anchors played a role in delaying the loss of composite action sufficiently to transfer larger forces to the CFRP, but they did not eliminate the bond-related limit state. Instead, the critical mechanism shifted toward the concrete substrate and anchor region. A similar effect of this behavior can also be observed in CP-1-A. There was no significant change or increase in ultimate load capacity; however, this did result in an increase in the maximum recorded displacement from 19.98 to 91.99 mm, with energy dissipation rising from 1097.49 to 5795.32 kN-mm. Since CP-1-A eventually experienced cover separation, the large increase in deformation is likely due to the plate staying attached and interacting with the beam section over an extended loading duration, rather than an increase in the peak flexural resistance of the section.

The TRM anchorage results further show that anchors are most beneficial when premature interface separation is the controlling mechanism and are less effective when matrix cracking, textile slip/rupture, or substrate failure governs. Anchorage increased the ultimate load in most directly comparable CTRM, BTRM, and GTRM pairs, but the associated ductility and energy changes were not consistently positive. In CTRM-3-A, damage shifted toward the end/anchorage region with flexural-shear cracking and TRM debonding; BTRM-3-A developed brittle concrete–cover detachment; and GTRM-3-A reached a 19.4% higher peak load than GTRM-3-NA but had lower ductility and energy dissipation. These changes are consistent with the anchor transferring a greater portion of the strengthening force into localized concrete regions, thereby delaying one debonding mechanism while potentially activating another. Conversely, the GTRM-1 pair showed a 5.2% lower peak load with anchors, confirming that anchorage cannot be assumed to provide a uniform strength increase. Given that each configuration was represented by one specimen, these trends are mechanistic interpretations of the observed failure patterns and should not be treated as statistically generalized anchorage effects.

### 4.5. Strain Distribution with Digital Image Correlation (DIC)

**Figure 13:**
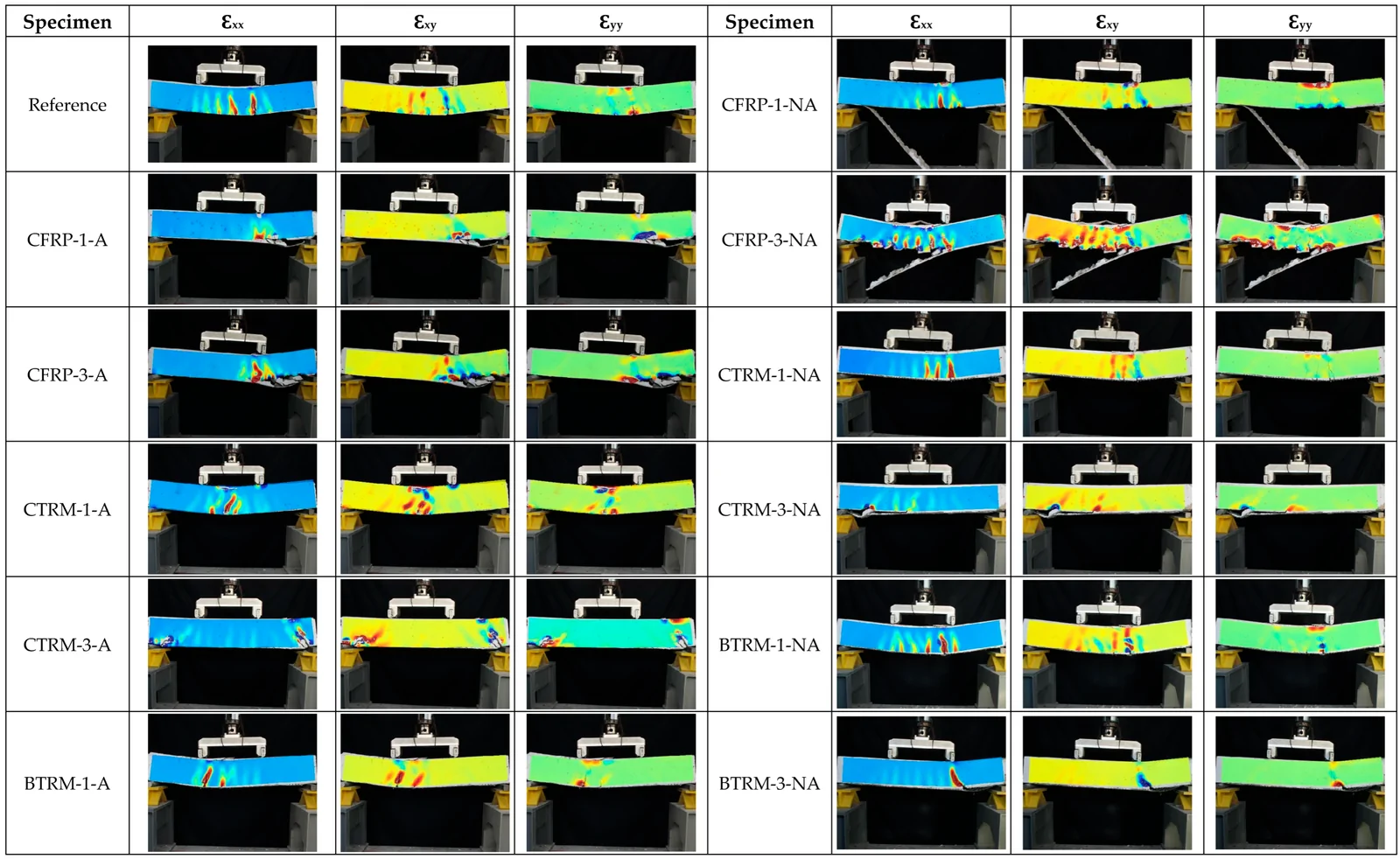

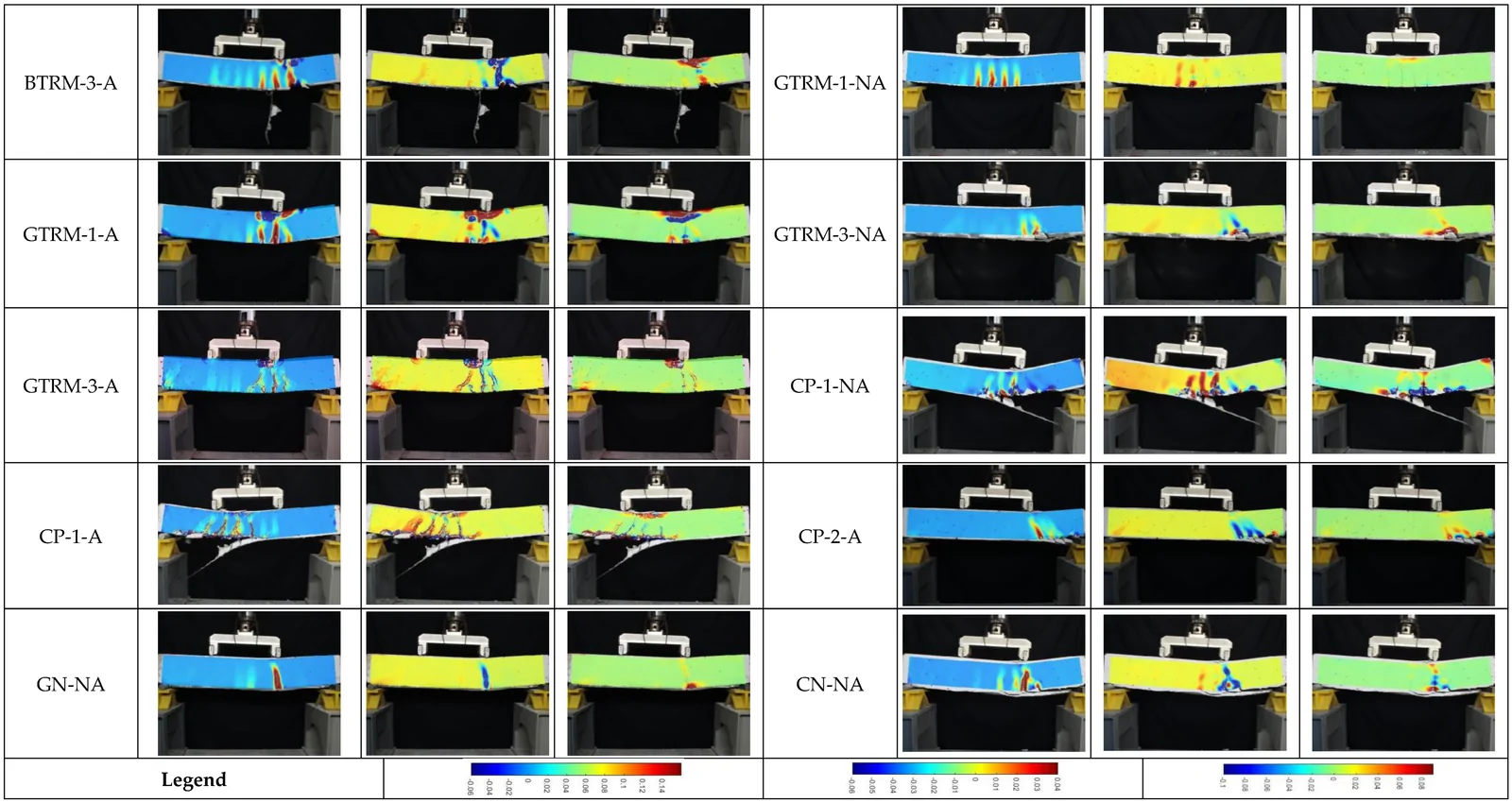
Strain distributions obtained with DIC.

The digital image correlation (DIC) method was used to evaluate the strain distributions of the test specimens during the final loading stage. The strain fields were examined in terms of three different strain components: ε_xx_, ε_xy_ and ε_yy_. To enable a consistent and objective comparison across all specimens, the DIC results were first analysed separately for each test specimen. Subsequently, the overall minimum and maximum strain limits were determined by taking into account the data obtained from all specimens for each strain component. Based on these lower and upper limits, a common strain range and colour legend were established, and the same scale was applied to all DIC images. The minimum and maximum strain limits are provided as a legend beneath the relevant figures, whilst the strain distributions were evaluated regionally, taking these defined common threshold values into account (Figure 13). Thanks to this approach, the localizations, distribution patterns and relative strain intensities of different specimens and reinforcement configurations could be compared under the same reference scale.

In the reference specimen, distinct concentrations of deformation corresponding to flexural cracks in the constant moment region are clearly visible. This indicates that the flexural cracks developed freely, without the limiting effect of any reinforcement system. Similarly, it can be seen that the ε_xy_ and ε_yy_ strain fields are also concentrated around the same central crack regions. This behaviour is consistent with the typical development of bending cracks expected in an unstrengthened reinforced concrete beam.

***CFRP specimens***

CFRP-1-NA: The longitudinal strain field appears narrower than the reference beam and is concentrated in a small number of cracks, indicating that the CFRP sheet has reduced crack spreading, resulting in damage developing at a single localized zone.

CFRP-1-A: Here, the strain is more localized, particularly in one area near the right half of the beam; this indicates that the anchorage has clearly improved force transfer, but overall, the response was governed by concentrated cracking rather than a widespread redistribution of strain.

CFRP-3-NA: This specimen revealed a large area of high ε_xx_, accompanied by clear and strong activity in both ε_xy_ and ε_yy_, as well as along the tension face, indicating intense interfacial action.

CFRP-3-A: The strain field was severe in this specimen, but less widespread than in CFRP-3-NA and more concentrated. This indicates that the anchorage was able to limit the overall spread of damage but did not prevent final localization.

***CTRM specimens***

CTRM-1-NA: The strain distribution here was greater in the middle zone compared with most CFRP specimens, indicating better crack dispersion.

CTRM-1-A: The field remains mainly central. However, the cracks appeared to be slightly more concentrated than those in CTRM-1-NA, indicating greater structural integrity with less widespread tensile strain.

CTRM-3-NA: In this specimen, most of the span remains at low strain. While the damage was concentrated locally, particularly near the end zone and the tension face, this was attributed to the strong crack control.

CTRM-3-A: In this specimen, the strain was concentrated at the ends or anchorage regions, particularly in ε_xy_ and ε_yy_. Here, the implied anchorage altered the load transfer path and made the final damage more pronounced at the ends.

***BTRM specimens***

BTRM-1-NA: The ε_xx_ field is spread over several cracks in the middle region. This indicates that the cracks were distributed symmetrically, with a relatively ductile flexural response.

BTRM-1-A: The strain here is still moderate and stable. There are fewer active cracks here than in BTRM-1-NA, so the response appears slightly more localized.

BTRM-3-NA: Strain activity is evident across a specific area of the right half of the beam with matching ε_xy_/ε_yy_ concentration, indicating a localized failure pattern.

BTRM-3-A: Several strain bands can be observed in this specimen, but they are mainly concentrated on one side of the beam.

***GTRM specimens***

GTRM-1-NA: This specimen shows several well-distributed ε_xx_ bands with relatively mild ε_xy_/ε_yy_ localization. This indicates effective crack propagation and a more stable response.

GTRM-1-A: The strain field appears more intense here, suggesting that multiple cracks formed prior to final localization. This pattern is consistent with a beam that was significantly deformed before failure.

GTRM-3-NA: In general, the strain distribution was more confined, with only one main active zone, indicating stronger control in terms of cracks. It is worth noting here that the distribution was less pronounced compared to the single-layer specimen.

GTRM-3-A: The specimen shows deformation near the loading area, particularly in ε_xy_ and ε_yy_. The strain map indicates localized cracking rather than widespread flexural damage.

***Carbon fiber plate specimens***

CP-1-NA: In this specimen, the strain concentrations were very high in all directions along the bond surface between the plate and the concrete in the central region, which explains the delamination of the concrete cover.

CP-1-A: The same mechanism was observed, but when compared to CP-1-NA, the strain field was more widespread, with many ε_xx_ bands. The use of anchorage contributed to delaying the debonding of the plate, resulting in a cracking process that was more resistant to damage.

CP-2-A: The strain here was located near the end of the plates instead of cracks spreading widely. Therefore, adding an extra strengthening layer did not effectively improve the uniformity of the strain field, as force transfer was the decisive factor here.

***NSM specimens***

GN-NA: The DIC map shows the presence of a single crack at ε_xx_, with some limited disturbances at ε_xy_ and ε_yy_, indicating more effective crack control and a more stable load transfer pattern.

CN-NA: Similar to specimen GN-NA, there is one principal crack that dominates the ε_xx_ field, with strong paired ε_xy_ and ε_yy_ concentrations at the same location.

Overall, the DIC strain fields indicate that the structural response was directly influenced by the degree of strain redistribution and the crack localization within the strengthened beams. Specimens exhibiting a range of strains along the x-x plane and well distributed across the constant moment zone demonstrated more stable flexural behavior and better crack control. In contrast, specimens characterized by intense localization of ε_xx_, ε_xy_, and ε_yy_ in a single crack zone or near the strengthening ends showed premature debonding or end-zone distress, despite in some cases attaining high peak loads. In this regard, the DIC results confirm that the effectiveness of the strengthening system depends not only on increasing the strength but also on maintaining compatible strain transfer and a distributed cracking pattern throughout the beam.

## 5. Conclusions

This study evaluated the bending behavior of reinforced concrete beams strengthened using externally bonded and near-surface-mounted systems, including carbon fiber-reinforced polymer (CFRP) sheets, carbon plates, textile-reinforced mortar with carbon, basalt, and glass textiles, and NSM bars with glass and carbon strengthening. The results of the experimental tests showed that the use of strengthening techniques improved the performance of RC beams compared with the control beam, bearing in mind that the degree of improvement depended primarily on the type of strengthening, the number of layers, and the anchorage condition. The maximum load of the reference beam was 60.01 kN, while all strengthened specimens exceeded this value, demonstrating the effectiveness of the strengthening techniques in improving the overall performance of reinforced concrete beams.

Among the investigated systems, the CFRP-strengthened specimens exhibited the largest ultimate-load increases, ranging from 54.94% to 84.04% relative to the reference beam. The CFRP-3-A specimen achieved the best performance in ultimate load capacity with a value of 110.44 kN, while the CFRP-3-NA specimen recorded the highest stiffness and energy absorption values of 18.83 kN/mm and 6509.34 kN-mm, respectively. These results demonstrate the effectiveness of CFRP sheets in enhancing flexural strength. Carbon plate strengthening increased the ultimate load by approximately 51.80–54.07% relative to the reference beam, with stiffness values ranging from 11.51 to 14.13 kN/mm, particularly when anchorage was provided, although its post-peak behavior appeared more sensitive to bond conditions. The use of the carbon TRM strengthening system plays an effective role in increasing load-bearing capacity, but its overall performance is inferior to that of CFRP sheets and carbon plates.

The TRM and NSM strengthening systems showed a lower increase in ultimate load capacity compared with carbon fiber-reinforced polymer (CFRP)-based systems but demonstrated greater ductility in several cases. The greatest ductility indices were obtained for GTRM-1-A and GTRM-1-NA, with values of 3.43 and 3.39, respectively, while BTRM and NSM specimens also exhibited considerably high deformability.

In general, the increase in the number of layers led to significant improvements in ultimate load and stiffness, particularly in the CFRP and CTRM groups. This indicates that increasing the number of strengthening layers effectively improves flexural behavior. However, increasing the number of layers did not result in a significant improvement in ductility; in fact, in some cases, it led to a stiffer response and reduced deformability. Similarly, the use of anchorage had a significant effect on increasing bond strength and ultimate load capacity, particularly in the CFRP and carbon plate groups. However, the use of anchors did not have a consistent effect on stiffness and energy absorption, suggesting that the anchorage system may alter the failure mode depending on the strengthening system used.

Overall, the study emphasizes that the choice of strengthening system should be based on the intended structural objective. If the main aim of strengthening is to achieve maximum strength and stiffness, then CFRP and carbon plates are the appropriate choice, whereas TRM and NSM strengthening are more effective when there is a need to improve ductility. Therefore, the study demonstrates that strengthening type, number of layers, and anchorage condition are all key parameters governing the flexural behavior of strengthened reinforced concrete beams.

## Figures and Tables

**Table 1:** Concrete mix design [32].

Targeted Concrete Compressive Strength Consistency W/C Dmax	25 MPa S3 0.529 11 mm
Material	Amount (kg)
0–4 Aggregate	1060
4–11 Aggregate	811
Concrete mixing water	150
Concrete mixing water-back	35
Cement (CEM-I-42,5-R)	350
Plasticizer	3.88
Fly ash	40

**Table 2:** Mechanical properties of steel reinforcement.

Properties	Diameter of 10 mm (Ø10 mm)	Diameter of 16 mm (Ø16 mm)
Yield Strength (MPa)	400	425
Ultimate Tensile Strength (MPa)	460	562
Modulus of Elasticity (GPa)	201	203
Type	Ribbed	Ribbed

**Table 3:** Typical Properties of FRP Textiles.

Carbon Textile Mesh Used in Strengthening with TRM *		Basalt Textile Mesh Used in Strengthening with TRM *	
Tensile Strength	255 kN/m	Mesh Tensile Strength	108 kN/m
Coated Weight per m^2^ (gr)	350	Coated Weight per m^2^ (gr)	350
Mesh Distance (mm × mm)	25 × 25	Mesh Distance (mm × mm)	25 × 25
Thickness (mm)	1.43	Fiber Elastic Modulus	80 GPa
Modulus of Elasticity (GPa)	235	Fiber Tensile Strength	4000 MPa
Elongation at Break (%)	1.7	Fiber Specific Gravity	2.7 g/cm^3^
Glass Textile Mesh Used in Strengthening with TRM *		Carbon Fiber-Reinforced Polymer SikaWrap-300C *	
Tensile Strength	70 kN/m	Dry Fiber Density	1.82 g/cm^3^
Coated Weight per m^2^ (gr)	315	Dry Fiber Thickness	0.167 mm
Thickness (mm × mm)	38 × 38	Dry Fiber Tensile Strength	4000 N/mm^2^
Modulus of elasticity (GPa)	72	Dry Fiber Modulus of Elasticity (tension)	230,000 N/mm^2^
Elongation at Break (%)	1.87	Dry Fiber Elongation at Break	1.7%
Carbon Fiber Plate *		Laminate Nominal Thickness	0.167 mm
Thickness	1.2 mm–1.4 mm	Laminate Tensile Strength (Average)	3500 N/mm^2^
Density	1.50 kg/lt	Laminate Modulus of Elasticity (Average)	225 kN/mm^2^
Tensile Strength	≥3000 MPa	Laminate Elongation at Break	1.56–1.59%
Modulus of Elasticity	≥165,000 MPa at rupture	Tensile Resistance (Average)	585 N/mm
Elongation at Break	1.4%	Tensile Stiffness (Average)	37.6 MN/m

* Mechanical properties are provided by the manufacturer.

**Table 4:** Typical Properties of Binding Materials.

Epoxy Resin *	
Applicable Ground Temperature	(+5 °C)–(+35 °C)
Concrete Adhesion	≥4.0 N/mm^2^ (Rupture from Concrete)
Bending Strength	≥40 N/mm^2^
Pressure Resistance	≥80 N/mm^2^
Tensile Strength	≥30.0 MPa
Repair Mortar *	
Application Temperature	(+5 °C)–(+35 °C)
Flexural Strength (EN 12190) [34]	≥7.0 N/mm^2^
Concrete Adhesion Strength (EN 1542) [35]	≥2.0 N/mm^2^
Compressive Strength (EN 12190)	≥45 N/mm^2^
Modulus of Elasticity (EN 13412) [36]	>20 GPa
Service Temperature	(−30 °C)–(+400 °C)

* Mechanical properties are provided by the manufacturer.

**Table 5:** Typical Properties of NSM Bars.

	Carbon Fiber Bar *	Glass Fiber Bar *
Fiber Content by Weight (%)	>75	>75
Fiber Content by Volume (%)	>65	>65
Tensile Strength (MPa)	>2080	>1000
Compressive Strength (MPa)	>1450	>550
Tensile Modulus (GPa)	>145	>38
Density (g/cm^3^)	>1.51	>1.80

* Mechanical properties are provided by the manufacturer.

**Table 6:** Description of strengthening details.

Spec. #	Group	Spec. Name	Comp. Strength of Concrete (MPa)	Details of Strengthening Materials	Number of Layers	Anchor Usage
1	Ref.	Ref.	25.29	Reference	-	-
2	1	CFRP-1-NA	25.66	carbon fiber-reinforced polymer (CFRP)	1	without
3		CFRP-1-A	24.49			with
4		CFRP-3-NA	26.24		3	without
5		CFRP-3-A	24.41			with
6	2	CTRM-1-NA	25.74	carbon textile-reinforced mortar (CTRM)	1	without
7		CTRM-1-A	25.58			with
8		CTRM-3-NA	24.72		3	without
9		CTRM-3-A	25.30			with
10	3	BTRM-1-NA	25.35	basalt textile-reinforced mortar (BTRM)	1	without
11		BTRM-1-A	24.47			with
12		BTRM-3-NA	25.26		3	without
13		BTRM-3-A	26.19			with
14	4	GTRM-1-NA	25.69	glass textile-reinforced mortar (GTRM)	1	without
15		GTRM-1-A	26.12			with
16		GTRM-3-NA	25.34		3	without
17		GTRM-3-A	25.05			with
18	5	CP-1-NA	24.33	carbon plates (CPs)	1	without
19		CP-1-A	25.53			with
20		CP-2-A	25.55		2	with
21	6	GN-NA	24.64	glass fiber bars, 16 mm (NSM)	-	-
22		CN-NA	25.91	carbon fiber bars, 16 mm (NSM)	-	-

**Table 7:** Experimental results.

#	Spec. Name	Max Load, Pmax (kN)	0.85 Pmax (kN)	Post-Peak Disp. at 0.85 Pmax (mm)	Disp. at Pmax (mm)	Ductility Ratio, μΔ (mm/mm)	Stiffness (kN/mm)	Max. Disp. (mm)	Energy (kN-mm)
1	Ref	60.01	51.01	46.07	37.7	1.22	1.59	46.23	2487.45
2	CFRP-1-NA	92.98	79.03	10.9	10.7	1.01	8.69	81.02	4816.25
3	CFRP-1-A	104.3	88.65	14.37	13.92	1.03	7.49	53.06	3279.86
4	CFRP-3-NA	101.52	86.29	8.45	5.39	1.56	18.83	87.02	6509.34
5	CFRP-3-A	110.44	93.87	8.36	8.29	1.01	13.32	51.34	3609.37
6	CTRM-1-NA	71.66	60.91	16.73	15.43	1.08	4.64	48.43	2738.11
7	CTRM-1-A	80.86	68.73	18.85	15.64	1.2	5.17	44.35	2663.08
8	CTRM-3-NA	93.17	79.19	14.69	14.39	1.02	6.47	64.05	3795.81
9	CTRM-3-A	98.01	83.3	22.91	13.24	1.73	7.4	23.39	1692.89
10	BTRM-1-NA	71.32	60.62	56.72	19.68	2.88	3.62	57.34	3493.4
11	BTRM-1-A	75.97	64.57	39.85	13.21	3.01	5.75	40.01	2590.99
12	BTRM-3-NA	83.35	70.84	19.03	18.48	1.02	4.51	32.89	2145.35
13	BTRM-3-A	89.82	76.34	25.21	21.96	1.14	4.09	59.29	3809.5
14	GTRM-1-NA	71.51	60.78	44.99	13.27	3.39	5.39	45.6	2725.74
15	GTRM-1-A	67.8	57.63	47.79	13.94	3.43	4.86	49.12	2730.4
16	GTRM-3-NA	79.94	67.94	18.61	11.98	1.55	6.67	64.3	3609.53
17	GTRM-3-A	95.41	81.09	22.02	20.07	1.09	4.75	34.25	2188.12
18	CP-1-NA	92.44	78.57	7.95	7.44	1.06	12.42	19.98	1097.49
19	CP-1-A	92.46	78.59	10.14	8.03	1.26	11.51	91.99	5795.32
20	CP-2-A	91.12	77.45	12.77	6.45	1.97	14.13	31.05	2037.53
21	GN-NA	66.73	56.72	32.2	10.33	3.11	6.46	32.39	1841.21
22	CN-NA	62.12	52.8	46.71	16.83	2.77	3.69	46.77	2557.9

## Data Availability

The original contributions presented in this study are included in the article. Further inquiries can be directed to the corresponding author.
